# Nanostructured Metals with an Excellent Synergy of Strength and Ductility: A Review

**DOI:** 10.3390/ma15196617

**Published:** 2022-09-23

**Authors:** Pengpeng Pu, Tijun Chen

**Affiliations:** State Key Laboratory of Advanced Processing and Recycling of Nonferrous Metals, Lanzhou University of Technology, Lanzhou 730050, China

**Keywords:** nanostructured metals, bimodal nanostructure, nanotwinned structure, gradient nanostructure, supra-nano-dual-phase structure, microstructure, mechanical properties, strengthening and toughening mechanisms

## Abstract

Nanocrystalline metals developed based on fine grain strengthening always have an excellent strength, but are accompanied by a drop in ductility. In the past 20 years, substantial efforts have been dedicated to design new microstructures and develop the corresponding processing technologies in order to solve this problem. In this article, the novel nanostructures designed for simultaneously achieving high strength and high ductility developed in recent years, including bimodal grain size distribution nanostructure, nanotwinned structure, hierarchical nanotwinned structure, gradient nanostructure, and supra-nano-dual-phase nanostructure, are reviewed. Based on a comprehensive understanding of the simultaneously strengthening and toughening mechanisms, the microstructures and corresponding processing techniques are mainly discussed, and the related prospects that may be emphasized in the future are proposed.

## 1. Introduction

Metals are indispensable structural materials in the construction of economy, aerospace, and defense fields [1]. With the rapid development of science and technology, modern industry has increasingly high requirements for excellent comprehensive mechanical properties of metals. The strengthening of metals has always been a hot topic, and the strength and ductility are the core criteria for measuring the quality of structural metals. Traditional strengthening methods such as solid solution strengthening, work hardening, and second-phase strengthening can greatly increase the strength, but always simultaneously damage the ductility in different degrees, that is, there is a strength–ductility trade-off dilemma [2]. Fortunately, fine grain strengthening can simultaneously increase the strength and ductility. For this purpose, Gileiter et al. first prepared nanocrystalline Fe using inert gas condensation in the early 1990s, and proposed the concept of nanocrystalline metals [3]. Nanostructured metals refer to the materials in which at least one dimension of microstructural unit (such as grain size or lamellar spacing) is on a nanometer-scale (1–100 nm) and that have a variety of morphologies, such as nanorods, nanowires, nanospheres, etc. [4,5]. Due to the unique nano-level grains and high-density grain boundaries, nanostructured metals thus exhibit many physical and chemical properties that are completely different from those of traditional coarse-grained metals [5]. In addition, their strength, hardness, wear resistance, and fatigue performance have been greatly improved compared with the corresponding coarse-grained counterparts [6]. Since then, nanostructured metals have become a research hotspot in the field of materials science.

However, when the grain size is decreased below nano-level, the strengthening is usually accompanied by a drop in ductility; as the grain size decreases, the strength increases significantly, while the fracture elongation sharply decreases and even exhibits brittle behavior [7,8,9,10]. The reason is that dislocation accumulation becomes impossible in such small grains, and dislocations emit from one grain-boundary segment and then disappear at another, leading to an extremely low work-hardening rate [11,12,13]. Indeed, most nanostructured metals have been found to exhibit zero work hardening [14,15]. As a result, the strength–ductility trade-off dilemma of nanostructured metals has become the bottleneck problem that hinders their development [16].

Therefore, the toughening of nanostructured metals has aroused the interest of researchers. In the recent 20 years, in order to improve their ductility, researchers have multilevel-constructed the composition, size, and distribution of different nanostructures, and some significant achievements have been made: (1) the bimodal structure is the introduction of a certain volume of micron-leveled coarse grains into the nanograined metal, and the resulting metal has the strength of the nanograined metal and the plasticity of the coarse-grained metal, i.e., good mechanical properties [17,18,19,20]; (2) by introducing nanotwinned (NT) structure inside the grains, the metal exhibits excellent mechanical properties, including high strength, plasticity, work-hardening rate, and fatigue performance, due to the relatively low interface energy and coherent characteristics of twin boundaries (TBs) [21,22,23,24]; (3) microstructure in which grain size, structure, and/or composition continuously change from nanometer-scale to micrometer-scale, i.e., a gradient nanostructure, has the performance advantages of each scale structure, so as to simultaneously realize strengthening and toughening [25,26,27,28]; (4) the supra-nano-dual-phase (SNDP) nanostructure consists of two phases with differences in structure or composition, i.e., heterogeneous two-phase nanostructure, and not only exhibits excellent mechanical properties, but also presents other functional properties, such as soft magnetic properties and thermal stability [29,30].

This article reviews the research progress of the above-mentioned new nanostructures, i.e., bimodal nanostructure, NT structure, gradient nanostructure, and SNDP nanostructure, in terms of preparation process, mechanical properties, and corresponding strengthening and toughening mechanisms. Based on these, the design ideas and processing methods of nanostructured metals with excellent synergy of strength and ductility are prospected.

## 2. The Bimodal Nanostructure

### 2.1. Original Intention

As mentioned above, nanograined metals, i.e., grain size *d* is on the order of nanometers (*d* < 100 nm), have obviously higher strength than their corresponding coarse-grained partners, but due to the large number of incoherent grain boundaries (GBs) and the limited dislocation storage capacity of the grains, their ductility is extremely low, which seriously limits their engineering applications [31,32]. Recently, it has been discovered that the introduction of coarse grains into the nanocrystalline matrix to form a bimodal grain size structure could obtain an excellent synergy of high strength and high ductility [33,34]. Generally, the essence of this structure is to utilize the high work-hardening capacity of coarse grains to improve the plasticity of nanograined metals without greatly damaging the strength [35].

### 2.2. Preparation Methods

The processing technology determines the size and distribution of grains in a bimodal nanostructure, which have important effect on the deformation mechanism and, thus, the mechanical properties of the structure. At present, there are three main methods for preparation of the bimodal nanostructures: thermomechanical processing route [19,20,36,37], powder metallurgy [38,39,40,41], and electrodeposition technique [42,43,44].

#### 2.2.1. Thermomechanical Processing Route

For the thermomechanical processing route, severe plastic deformation (SPD) is first utilized to obtain nanograins, and then an annealing treatment is used to achieve coarse grains by partial coarsening [19]. A strong large strain is usually introduced during SPD at low temperatures [45]. Such deformation can inhibit dynamic recovery so that nanograins are obtained; in addition, it allows more energy to be stored inside the metals, making recovery and recrystallization easier during subsequent annealing. The annealing temperature is usually lower than the recrystallization temperature. In this case, the grain growth temperature of nanograins is much lower than the recrystallization temperature of the corresponding coarse grains, and thus, only partial coarse grains are obtained through coarsening, while the work hardening and residual stress are eliminated.

**Figure 1:**
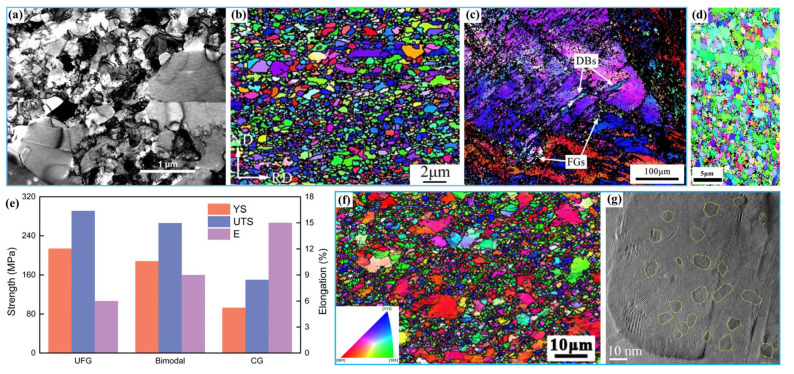
(**a**) Transmission electron microscopy (TEM) image of bimodal nanostructure Cu [18]; (**b**) electron backscatter diffraction (EBSD) inverse pole figure (IPF) of bimodal-structured steel [20]; (**c**) EBSD IPF of Al–Mg alloy after high strain rate rolling (DBs and FGs refer to deformation bands and fine grains, respectively) [19]; (**d**) EBSD IPF of a bimodal Al6063 alloy [41]; (**e**) the mechanical properties of ultrafine-grained structure, bimodal structure, and coarse-grained structure Al6063 alloys, “E” represents the strain to fracture; (**f**) EBSD IPF of a bimodal structure Ni (the inset shows the standard IPF color code) [42]; (**g**) TEM image of an ultrafine-grained structure in which nanoscale Ni domains are distributed (the area circled in yellow refer to nanoscale Ni domains) [43].

Wang et al. [18] prepared a bimodal nanostructure in Cu by this method for the first time, in which 25 vol.% micrometer-sized grains (1–3 μm) were embedded inside an ultrafine-grained matrix (less than 300 nm), as shown in Figure 1a. The resulting Cu possessed a tensile yield strength (YS) of ~325 MPa and a fracture elongation of ~65%. Subsequently, in order to improve the elongation (~4.1%) of an ultrafine-grained low-carbon steel composed of ferrite and martensite [36], Wang et al. [37] prepared a bimodal structure by appropriate annealing of a cold-rolled steel. The microstructure was composed of fully recrystallized ferrite with ~60 vol.% ultrafine grains (~0.7 μm) and ~40 vol.% coarse grains (~5.0 μm) in which nanoscale carbides (~60 nm) were dispersed. The tensile YS, ultimate tensile strength (UTS), and uniform elongation increased from ~658 MPa, ~672 MPa, and ~4.1% to ~663 MPa, ~788 MPa, and ~17.8%, respectively. Based on the research of Wang et al. [36], in order to further refine the grains and obtain more excellent mechanical properties, Niu et al. [20] prepared an austenitic stainless steel (SS) with a bimodal nanostructure by using two cold-rolling and annealing processes (Figure 1b). The second cold-rolling and annealing was performed on a bimodal microstructure obtained from the first cold-rolling and annealing process. Both the coarse and fine grains were further refined, and the final microstructure was composed of ~80% nanograins (~50 nm) and ~20% micrometer grains (~2 μm). The resultant steel had superior comprehensive mechanical properties with YS of ~1.2 GPa and uniform elongation of ~45.3%. Compared with the bimodal-structured steel obtained by single cold rolling and annealing, the YS was increased by 65% while the elongation was only sacrificed by 16%.

In contrast to the traditional multipass rolling process, Li et al. [19] obtained an Al–6.5Mg alloy with a bimodal structure by one-pass high strain rate rolling at 320 °C, in which the coarse and ultrafine grain sizes were ~2.1 μm and ~366 nm, respectively. The microstructure was composed of ultrafine grains, deformation bands, and deformation regions lacking dynamic recrystallization, and the fine grains were primarily detected along the strain-induced deformation bands related to high strain rate, as shown in Figure 1c. Compared with the traditional rolled Al–6.5Mg alloy, the high strain rate rolled sample exhibited significant grain refinement and an increase in deformation band number, and its YS, UTS, and elongation were increased from 251 ± 2 MPa to 262 ± 6 MPa, 365 ± 2 MPa to 387 ± 9 MPa, and ~17.1% to ~23.3%, respectively.

Although the thermomechanical processing route can produce bimodal nanostructures simply and efficiently, it is found that the resultant grain size is generally in micrometer-scale due to the limited saturation of plastic deformation in crystals. More importantly, the magnitude of deformation force always decreases sharply from the surface to the interior, so the resultant nanostructure is generally concentrated on the surface and its thickness is quite limited. In addition, the distribution of the coarse and fine grains is difficult to control accurately. Finally, this technology is only applicable to metals with good plasticity, resulting in significant limitations on the types of metals that can be prepared.

#### 2.2.2. Powder Metallurgy

In order to solve the problems of the thermomechanical processing route mentioned above, powder metallurgy was developed [38]. In the process, nanocrystalline powders are first obtained by using high-energy ball milling or inert gas condensation, and then mixed with unmilled powders having coarse grains, and finally the mixture is pressed, hot isostatic pressed, or spark plasma sintered. The mass (volume) ratio and sizes of coarse grains and nanocrystalline powders can be reliably controlled in a wide range, so this method has become a unique and feasible fabrication technique.

Witkin et al. [38] used low-temperature ball milling of coarse-grained (~4 μm) Al–7.5Mg alloy powder to achieve powders with a grain size of ~20 nm, and then uniformly mixed them with the unmilled powders. Finally, the mixture was consolidated and extruded to obtain a bulk nanostructured Al alloy with bimodal grain sizes. Compared with the all-cryomilled sample, the elongation of the resulting sample was improved by 71% and 286%, respectively, as the volume fraction of the unmilled powders was increased from 15% and 30%, while the YSs were decreased by 1.7% and 13.6%, respectively. In order to avoid the brittleness of nanograined pure Fe, a bimodal nanostructure containing certain fractions of coarse grains (>1 μm), nanograins (<100 nm), and nanoscale oxides (~10 nm) was produced by mechanical milling and subsequent spark plasma sintering, and it had a UTS of ~1.5 GPa and a total elongation to fracture of ~15%. The bimodal nanostructure rendered a large proportion of such high strength in addition to the strengthening role from the nano-oxides [39]. In a similar study from Witkin et al. [38], the strength of a bimodal structured Fe also decreased slightly as the volume fraction of coarse grains increased, but the plasticity increased continuously.

To verify the idea that the ductility of nanocrystalline Al alloys can be improved by incorporating a coarse-grained component, a series of bimodal nanostructured 5083 Al alloys were prepared by consolidation of blended mixtures of cryomilled and unmilled powders, in which the grain sizes in the ultrafine-grained regions and the coarse-grained regions were ~200 nm and ~1 μm, respectively [40]. With the increase in volume fraction of the coarse grains from 0% to 50%, the uniform elongation was increased by ~330% with a small sacrifice of ~12.8% in UTS. Similarly, Shakoori et al. [41] prepared an Al6063 alloy with bimodal structure by high-energy ball milling and subsequent hot extrusion, and found that both the ultrafine grains (80 nm–1.4 μm) and coarse grains (1.8 μm–4.1 μm) were uniformly distributed, as shown in Figure 1d. Compared with the coarse-grained and ultrafine-grained structures, it was found that the bimodal structure achieves an attractive balance of strength and ductility (Figure 1e).

Based on the above discussion, it can be concluded that the powder metallurgy can quantitatively control the volume fraction of coarse grains by altering the amount of unmilled powders. In this case, the strength achieved from ultrafine grains is coupled with the ductility provided by coarse grains, and a good synergy of strength and ductility can be obtained [40]. However, since this method is a synthetic method, it is easy to introduce impurities and pores during powder mixing, causing nonintrinsic low plasticity. In addition, since nanocrystallines are effortless to grow during the sintering process, and it is difficult to accurately control their size [46], the obtained metals always required later heat treatment and/or plastic processing, such as hot extrusion, cold rolling, etc., resulting in the cumbersome processing steps and difficulties in processing components with complex shapes.

#### 2.2.3. Electrodeposition Technique

In order to obtain bimodal nanostructured metals with controllable grain sizes and no impurities, the electrodeposition technique was developed. During the electrodeposition process, the metal ions in the solution near the cathode are discharged and deposited onto the cathode through electrocrystallization, generating lots of crystal nuclei on the deposited surface. The grain sizes of the deposited layer are related to the grain growth rate during electrocrystallization, and dense bimodal nanostructured metals can be synthesized by adjusting the current density, additives, and other parameters [47].

Zhang et al. [42] prepared a bimodal nanostructured Ni by using the direct current electrodeposition technique; as shown in Figure 1f, the microstructure shows a grain size of ~450 nm in ultrafine-grained regions and ~1.15 μm in coarse-grained regions. Compared with those of the ultrafine-grained Ni, the uniform elongation of the nanocrystalline Ni was increased from ~3% to ~13.1% while the UTS was only decreased from ~890 MPa to ~847 MPa.

The bimodal structure with ultrafine grains and coarse grains can well improve the strength-to-ductility ratio of metals, but can it possess more excellent performance? A bimodal nanocrystalline Ni with 150–300 nm coarse grains surrounded by 10–30 nm nanograins was fabricated by Gu et al. [44] utilizing direct current electrodeposition, exhibiting a high UTS of ~1.2 GPa and a relatively high ductility of ~7.5%. It also showed a better ductility than the unique nanograined Ni. In order to further adjust the grain size and distribution of the two constituents, a bimodal Ni with a broad grain size distribution was fabricated via the surfactant-assistant electrodeposition technique, in which there were ~35 vol.% ultrafine grains with sizes of 80–160 nm and ~45 vol.% nanograins with sizes of 10–40 nm, and a UTS of ~1.73 GPa with an elongation to failure of ~8.3% was realized [48]. Furthermore, Wu et al. [43] constructed a bimodal nanostructured Ni in which a volume fraction of 2.4% ~7 nm nanoscale domains was dispersed in ~150 nm ultrafine-grain matrix by pulsed electrodeposition, as shown in Figure 1g. The resultant Ni had a YS of ~1.3 GPa and a uniform elongation as large as ~30%, which were equivalent to and 16 times that of the uniform nanograined Ni, respectively.

Electrodeposition is a method to efficiently prepare nanostructured metals with bimodal grain size using an electrochemical process. It has the advantages of controllable microstructures, simple equipment, and no impurity introduction. However, the thickness of the achieved sample is generally only in the order of microns, and the production efficiency is quite low, which is only suitable for basic theoretical research and cannot be used in large-scale production in engineering.

### 2.3. Strengthening and Toughening Mechanisms 

**Figure 2:**
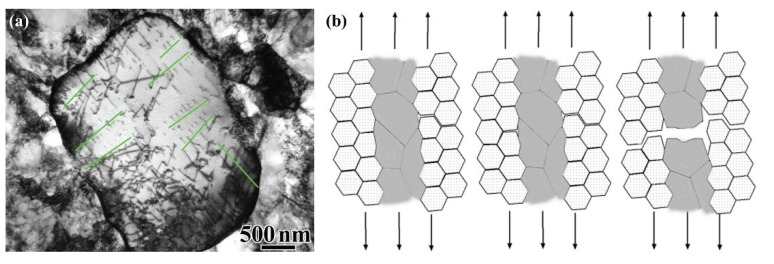
(**a**) A coarse grain surrounded by ultrafine-grained matrix in a bimodal structured Ti (dislocations pile-ups are marked by green lines) [51]; (**b**) the model of crack propagation in the bimodal structure (the arrows represent the directions of stress) [40].

Numerous studies have found that the high strength of the bimodal nanostructures is attributed to the nanocrystalline matrix, and the excellent ductility is ascribed to the extra strain hardening originating from the multidirectional stress states and strain gradients caused by the performance differences between the coarse and fine grains, and the abundant geometrically necessary dislocations (GNDs) generated within the coarse grains [40,49,50]. Wu et al. studied the tensile deformation mechanism of a bimodal nanostructured Ti, and proposed that the coarse grains first plastically deformed due to lower YS than the nanocrystals, but their morphologies did not obviously change because of the restriction from their surrounding nanograins. As a result, the coarse grains carried more plastic strain than the nanograins, and this phenomenon was called strain partitioning [51], which made a larger strain gradient near the interface between the two constituents. In order to accommodate the strain gradient, abundant GNDs then accumulated in the coarse grains close to the interface (Figure 2a), resulting in long-range back stresses and causing the coarse grains to be work-hardened to achieve an intermediate strength between the two kinds of grains. The back-stress work hardening helped to prevent necking during the tensile process, and thus improved the ductility of metals, resulting in a high ductility as well as a high strength [51,52].

To more clearly clarify the roles of the nanograins and coarse grains in the deformation process, a model of crack blunting combined with the concept of delamination was established by further studies on the fracture process of Al alloys [40,49]. As shown in Figure 2b, voids first initiate, grow into microcracks, and propagate along GBs in the nanograined regions under tensile load. As the cracks meet coarse grains, their propagation is then retarded through blunting of the crack tips or by interface delamination between the coarse-grained and fine-grained regions. With the further increase of the applied stress, necking occurs and cavitation generates within the coarse-grained regions, and, finally, dimples on the coarse-grained regions or delamination of the interfaces form on the fracture surface [40]. Both the delamination and necking would consume significant energy, resulting in an improved ductility. That is, the toughening of the bimodal nanostructures was attributed to the delay of the coarse grains in propagation of cracks that initiate in the fine-grained regions. This toughening mechanism was also called “the ductile-phase toughening” [53].

Zhu et al. [54] further explored the effect of nano- or microscale cracks on the mechanical properties of bimodal nanostructured metals during the deformation process through establishing a theoretical model with the aid of a modified mean field approach, and found that the cracks generated in the nanostructure matrix could obviously release stress concentration and significantly improve plastic deformation ability of the metals. At the same time, the cracks led to an increase of dislocations agglomerated along GBs, and then back stresses were enhanced, which gives rise to an additional contribution to strain hardening. In addition, the simulation results from a bimodal-structured Ni by this model showed that the strain gradient acts on the yield stress, while the back stress contributes to the strain hardening [54,55]. The mechanical properties of various metals with bimodal structures were simulated by using this model, and the results were in good agreement with the experimental ones [18,56,57].

In brief, the cracks are first generated within the nanograined regions and propagate into the coarse-grained regions, but find it difficult to further develop due to the excellent work-hardening ability of the coarse grains, so the overall plastic instability of the metals is suppressed, resulting in a good plasticity. When the work-hardening capacity of the coarse grains is exhausted by cracks, the crack propagation can no longer be inhibited, and the homogeneous plastic deformation of the metals is terminated, leading the metals to fracture.

### 2.4. Summary and Prospect

The above results indicate that the bimodal structures can significantly improve the ductility of nanostructured metals, and the methods used for preparing such structures are relatively simple. However, this strategy still faces many problems, as follows:

(1) The “reproducibility” of such structures is not ideal by using the existing preparation methods. The most commonly used methods, such as the thermomechanical processing route and the powder metallurgy, make it difficult to accurately control the grain size, grain morphology, and spatial distribution of the two constituents, due to the inhomogeneity of plastic deformation during plastic processing or ball milling and the temperature nonuniformity during annealing or consolidation [52,58]. These make bimodal nanostructures not so “reproducible” that overall deformation process can be accurately predicted and modeled.
(2) The influences and corresponding mechanisms of microstructure parameters on the mechanical properties are not completely understood. Although previous studies have analyzed the effect of volume fraction of the coarse-grained regions on ductility of Al–7.5Mg alloy, Fe, and 5083 Al [38,39,40], those of the other microstructure parameters, such as the grain sizes, mechanical behaviors, spatial distributions, and more detailed volume–fraction ratio of the two constituents, and the optimum parameters have not been comprehensively clarified. The main reason is that the ideal microstructure cannot be achieved due to the limitations of the processing techniques mentioned above.
(3) The trade-off between strength and ductility still exists sometimes. As for the bimodal structured austenitic steel [20] and Al–Mg alloy [38] prepared using thermomechanical processing and powder metallurgy method, respectively, they showed an increase in strength but a decrease in ductility compared to the corresponding coarse-grained counterparts, which are different from the currently recognized strengthening and toughening mechanisms and need to be further clarified.

## 3. The Nanotwinned Structure

### 3.1. Design Concept

It is known that TB is a special type of coherent GB, of which the atoms arrangement on both sides is mirror-reflected relative to the interface and has a high degree of symmetry, and has higher stability than GB due to its lower boundary energy, and thus can be taken as a promising strengthening boundary [22]. However, the strengthening effect of micro- or sub-microscale twin lamellae was not obvious, while that of nanoscale twin lamellae was quite significant [21]. Therefore, NT metals have been studied extensively in recent years [47,59,60,61,62,63]. The nanoscale TBs could not only hinder the movement of dislocations more effectively than the conventional high-angle GBs, leading to a higher YS [64,65], but also could absorb dislocations to withstand larger plastic deformation, resulting in higher ductility. That is, the NT structure is an effective way to simultaneously obtain high-strength and high-toughness metals [22,23,64,65,66].

In addition to the most common paralleled nanotwins, Lu et al. proposed a spatially oriented nanotwin structure, named hierarchical nanotwinned (HNT) structure [2]. The nanotwins with a single distribution orientation have two-dimensional barriers inside grains and can only hinder the dislocation movement in limited direction, while the HNT structure has multiorientation nanotwins, which construct a complex spatial multidimensional structure, and thus, possess a better barrier effect on dislocation movement in terms of more directions [2,67,68,69].

### 3.2. The Single-Order NT Structure 

#### 3.2.1. Preparation of Nanotwins

In general, the NT structure can only be generated in metals with low stacking fault energy (SFE), such as Cu [70], Ag [71], Ni [72], and 316L SS [73,74]. The preparation methods can be divided into two categories according to the formation mechanism of nanotwins. The first category is the SPD method for deformation twins, such as dynamic plastic deformation (DPD) [75,76,77]; the employed high strain rate and/or low temperature during deformation can effectively suppress dislocation motion and promote twin deformation. The other one is the deposition method for growth nanotwins, such as physical vapor deposition (PVD) [78,79] and electrodeposition [59,80,81,82]. The used high current density or deposition rate accelerates the nucleation, and the massive nucleation is accompanied by forming high-density GBs, which leads to higher stresses. When the current or deposition stops, nanoscale growth twins are then formed driving by stress relaxation [83].

(1) DPD method

In general, the formation of deformation twins is affected by internal and external conditions [84]. The internal conditions refer to the metal intrinsic parameters, such as grain size, grain orientation, and SFE, and the external ones include the processing parameters, such as deformation degree, strain rate, deformation temperature, etc.

As the most important intrinsic parameter, the SFE is closely related to the twinning ability of metals. The energy required to form a stacking fault (SF) per unit area is called SFE [85]. A lower SFE means that SFs are more stable, and thus deformation twins can be more easily generated during plastic deformation [86]. Dislocation slip and deformation twinning are the two main competing mechanisms for plastic deformation [84]. When the critical shear stress of dislocation slip is smaller than that of twinning, the dislocation slip is easier to operate, and on the contrary, the deformation twinning dominates the plastic deformation. According to the study from M. A. Meyers et al. [84,87], the critical shear stress (${\bar{\tau }}_{T}$) of twin nucleation is expressed as

(1) $${\bar{\tau }}_{T}=K\sqrt{\gamma_{\mathrm{SF}}/\mu b}$$

where K is a constant, $\gamma_{\mathrm{SF}}$ is the SFE, μ is the shear modulus, and b is the Burgers vector. It was found that the smaller the SFE $\gamma_{\mathrm{SF}}$, the smaller the critical shear stress ${\bar{\tau }}_{T}$, and the easier the twinning.

The main external conditions included strain rate and deformation temperature [86]. Increasing strain rate and reducing the deformation temperature can significantly increase the critical shear stress of dislocation slip, but have little effect on that of twinning, resulting in an obvious improvement of deformation twinning ability [47,86,88,89]. The effects of strain rate (ε) and deformation temperature (T) on deformation twinning can be coupled into a parameter Z, which can be expressed as [90]

(2) Z=ε·exp(Q/RT)

where Q is the deformation activation energy, and R is the gas constant. It is found that reducing the deformation temperature and increasing the strain rate can increase the parameter Z, and the volume fraction of twins was thereby increased and the twin thickness (i.e., the spacing between two adjacent TBs, hereafter referred to as λ) was reduced [91]. Therefore, the SPD method with high strain rate and low deformation temperature is beneficial for forming high volume fraction of small-spacing nanotwins in metals with low SFE.

According to the above regulation, researchers have developed a plastic deformation technique in high strain rate (10^2^–10^3^ s^−^^1^) and/or a low deformation temperature (77 K), i.e., DPD method [92]. The typical process is to use a hammer at the top of the sample to perform high-speed impact on the sample from a certain height, as the aim is to achieve a dynamic plastic deformation [84]. The λ can be controlled through adjusting the deformation temperature, deformation rate, and deformation pass [45]. The resulting microstructure often shows high-density dislocations accumulated along TBs, which may lead to reduction in elongation, so a subsequent heat treatment is needed to increase the elongation through reducing dislocations. That is, the annealing for DPD was to reduce the dislocation density, but not to obtain coarse grains through partially coarsening fine grains for the thermomechanical processing to prepare bimodal nanostructure.

**Figure 3:**
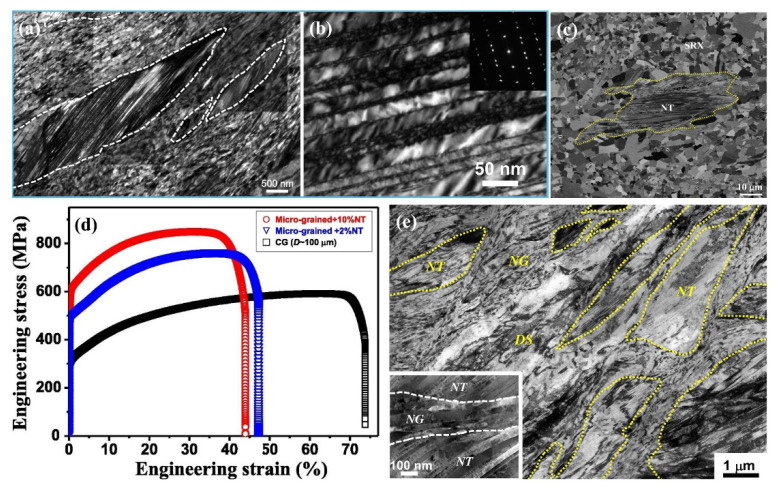
(**a**,**b**) TEM image of DPD 316L austenitic SS (the NT bundles are enclosed by white dash lines), inset in (**b**) is the corresponding selected area electron diffraction (SAED) pattern [73]; (**c**) scanning electron microscopy (SEM) image of the DPD 316L austenitic stainless after annealed (the NT and SRX refer to nanotwins and static recrystallization grains, respectively) [74]; (**d**) tensile engineering stress–strain curves of austenite 316L steels containing 10 and 2 vol.% of NT-γ grains [93]; (**e**) bright-field TEM image of DPD Cu–Ag sample, inset is the scanning transmission electron microscope-high angle annular dark field (STEM-HAADF) image of the DPD sample, the labeled NT and the areas circled by yellow dotted lines, NG, and DS refer to nanotwins, nanograins, and dislocation structures, respectively [65].

Figure 3a shows the microstructure of a 316L austenitic SS, in which NT bundles (marked by dashed circles) are embedded in nanograined matrix prepared by DPD method with plastic strain of 1.6 at ambient temperature [73], and there are high-density dislocations located at TBs (Figure 3b). After annealing at 730 °C, the YS and UTS of the sample increased from ~275 MPa and ~585 MPa of the coarse-grained counterpart to ~1020 MPa and ~1115 MPa, respectively, while the total elongation reached ~18%. Similarly, Yan et al. [74] also prepared a bulk nanostructured 316L austenitic SS by DPD with a high strain rate of 10^2^–10^3^ s^−1^ at ambient temperature, and its microstructure consisted of nanograins (NT-γ grains) embedded with deformation nanotwins (marked by NT in Figure 3c). The average grain size, λ, and volume fraction of NT bundles were ~33 nm, ~20 nm, and ~24%, respectively. The annealed sample had a superb UTS of ~1.0 GPa and an elongation to failure of ~27%. Lu et al. [93] further studied the role of NT-γ grains through comparing the mechanical properties of 316L SS with different amounts of NT-γ grains, and the YS was increased by ~25% when the volume fraction of NT-γ grains was increased from 2% to 10% (Figure 3d). The superior UTS was originated from the NT-γ grains, which possessed a very high YS of ~2 GPa, and the considerable tensile ductility contributed to the relatively high work-hardening rates [94]. In addition, the available investigations also showed that the strength of NT structures increased with increasing strain during DPD while the elongation decreased due to the increased nanotwins and dislocation density; an opposite result was achieved as the annealing temperature rose due to the reduction in dislocation density and moderate grain coarsening [73,95].

In addition to the deformation twins prepared in the steels, Zhang et al. [65] prepared a mixed nanostructure consisting of nanotwins, dislocations, and nanograins in a Cu–5Ag alloy via means of DPD at liquid nitrogen temperature (Figure 3e), and this nanostructure exhibited a YS of ~527 MPa, a UTS of ~549 MPa, and a total elongation of ~21.8%. Adding a small amount of Ag into Cu can significantly reduce SFE and, thus, the thickness of nanotwins [74,96,97], so the nanotwins in the Cu–Ag alloy were finer than those in the Cu alloy [98]. In addition, annealing treatment decreased the volume fractions of both nanograins and NT bundles, but the decrement of the former (~90%) was obviously larger than that of the latter (~43%), due to the lower thermal stability of GB than TB [99] (which originated from the larger excess energy of GB (0.625 J/m^2^) than TB (0.02–0.04 J/m^2^) [65]).

(2) PVD method

PVD is a process that uses physical methods to convert solid metals into gaseous atoms or ions under high vacuum conditions, and then they migrate and deposit on a substrate surface to form a solid film [47]. At present, magnetron sputtering is the main PVD method for successful preparation of high-density growth nanotwins, and its basic principle is that Ar ions inside the chamber are accelerated to the cathode target under the action of electric field force, the target atoms are blasted out of the target metal to sputter, and the target atoms or molecules are deposited on the substrate under the action of electromagnetic fields to form thin films [71,100].

**Figure 4:**
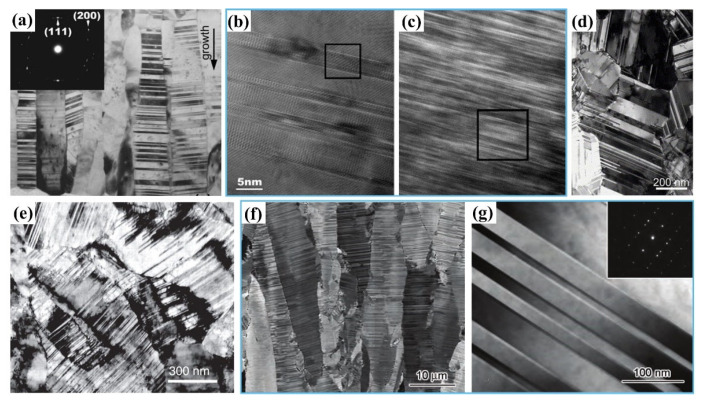
(**a**) TEM micrograph of Cu foils with NT structure by magnetron sputtering [70]; (**b**) high-resolution TEM (HRTEM) image of nanotwins in Cu (regular nanoscale twins are shown in the black square) [101]; (**c**) HRTEM image of twins in 330 SS (extremely fine scale twins are shown in the black square) [101]; (**d**) TEM image of NT Cu prepared by pulsed electrodeposition with λ of 15 nm at current density of 70 mA/cm^2^ [102] and (**e**) 4 nm at current density of 300 mA/cm^2^ [60]; (**f**) SEM image of direct current electrodeposited NT Cu [103]; (**g**) TEM image of NT Cu and the SAED pattern (the inset) [103].

Zhang et al. [70] synthesized a Cu foil with an average λ of ~5 nm via magnetron sputtering, and the deposition rate varied in the range of 0.5–2.0 nm/s; the microstructure was composed of columnar grains with an average column diameter of ~43 nm and exhibited a <111> fiber texture, and the long axis direction was consistent with the growth direction (Figure 4a). Particularly, the columnar nanograins contained numerous nanotwins, and the twin planes were arranged in a preferred direction perpendicular to the growth direction. The sample exhibited a high UTS of ~1.2 GPa, ~3 times higher than that of the nanograined Cu, and a uniform elongation of ~2%.

In the magnetron sputtering method, the deposition rate was the most important parameter for determining the density of nanotwins, besides the SFE of metals [82,104]. The study from Anderoglu showed that the average column diameter of columnar grains in an NT Cu sample decreased from 146 nm to 70 nm and the average λ decreased from 16 nm to 7 nm with increasing the deposition rate from 1 nm/s to 4 nm/s [105]. This was consistent with the conclusion drawn from the thermodynamic analysis of twin nucleation in NT Cu [70] and Cu/330 SS multilayer films [101]: the higher the deposition rate, the higher the nanotwin density and the smaller the laminar spacing λ. Meanwhile, as indicated by Figure 4b,c, the SFE of 330 SS (10–20 mJ/m^2^) was lower than that of Cu (45 mJ/m^2^), so the twin density in the 330 SS layer was much higher than that in the Cu layer, and the λ was far smaller. That is, high-density nanotwins, similar to the NT structure prepared by DPD method, were also easy to generate in low-SFE metals during magnetron sputtering [101].

(3) Electrodeposition method

Electrodeposition is a typical electrochemical process, in which a metal layer is generated on a cathode surface in an electrolytic cell through reduction reaction and electrocrystallization of metal ions [106]. This method can increase the probability of atoms stacking through precisely controlling the electrodeposition parameters to obtain high density of growth nanotwins [83].

In the process of pulsed electrodeposition, new GBs can be formed in between the pulse intervals, which can effectively prevent the grains from coarsening with time, and the microstructure is in an equiaxed NT structure [59]. Meanwhile, the current density has a significant effect on the λ. Figure 4d,e present the microstructure of an equiaxed NT Cu prepared by pulsed electrodeposition, which shows that the average λ reduced from 15 nm to 4 nm as the current density increased from 70 mA/cm^2^ to 300 mA/cm^2^ [60]. The TBs in each grain are parallel to each other, but the grains’ orientations were randomly distributed [47]. Compared with pulsed electrodeposition, direct current electrodeposition usually has a smaller current density and better current continuity, which enables continuous growth along the deposition direction, and the prepared sample had a columnar NT structure generally [85]. As the λ decreases, both the YS and elongation of the NT metals are simultaneously increased. The copper sample possesses a tensile YS of ~900 MPa and UTS of ~1068 MPa, respectively (~10 times higher than UTS of the coarse-grained counterpart), and a considerable elongation to failure of 13.5% at the average λ of 15 nm; but as the average λ was reduced to 4 nm, the strength exhibited a softening behavior, and the YS and UTS were decreased to ~310 MPa and ~700 MPa, respectively, while the uniform elongation was increased to ~30% [107].

However, not all NT metals have a softening effect. A continuous strengthening effect was observed in NT Ni prepared by direct current electrodeposition as the average λ decreased from 81 nm to 2.9 nm, achieving an ultrahigh strength of 4.0 GPa and a ductility of 2.5% at λ of 2.9 nm, which was 12 times stronger than that of the coarse-grained counterpart [108]. Figure 4f gives the cross-sectional SEM image of an NT Cu prepared by direct current electrodeposition. It shows that most of the grains are in a columnar morphology along the growth direction, and each grain contains a high density of TBs, which are mostly parallel to the growth plane [103]. In addition, most of the TBs grow well and penetrate the entire grain, and the twin thickness is on the order of nanometers (Figure 4g). Meanwhile, the TBs are straight and clear, and the twin lamellae are relatively clean with few dislocations. As shown by the inset in Figure 4g, the TBs are Σ3 coherent boundaries and the twin planes are (111) planes, which are arranged preferentially along the growth direction within the columnar grains, leading the columnar grains to show a (111) texture [109].

Based on the above discussion, it is found that bulk NT metals can be successfully fabricated by the DPD method. However, the achieved microstructure is a mixed nanostructure composed of high-density dislocations, nanograins, and TBs, and the volume fraction of the NT structure is relatively low, i.e., it is not a “clean” NT structure. In contrast, the NT structures achieved by electrodeposition and PVD methods are completely composed of growth twins, the grain size and the λ are smaller, and there are fewer defects at TBs, which supplies one ideal NT structure for investigating the strengthening and toughening mechanisms of such a structure. However, most of the NT film samples prepared by the two deposition processes mentioned above are only tens to hundreds of microns in thickness due to the low deposition efficiency, which makes it difficult to realize engineering applications. Although the DPD method is simple and easy to operate, and the thickness of the resulting NT samples always can reach millimeter level, the achieved microstructure is mixed and the volume fraction of nanotwins is low.

#### 3.2.2. Strengthening and Toughening Mechanisms

It can be found that the NT metals also have excellent strength–ductility synergy and high work-hardening rates, and the existing studies were basically focused on the FCC metals such as Cu, Ag, Ni, etc. [60,65,108]. Their excellent mechanical properties are attributed to the unique interaction behaviors between twins and dislocations in stressed state [92,102]. More importantly, the twin thickness (λ) and orientation have significant effect on the deformation mechanism and, thus, the mechanical properties of the NT metals [60].

(1) Effect of twin orientation on deformation mechanisms

**Figure 5:**
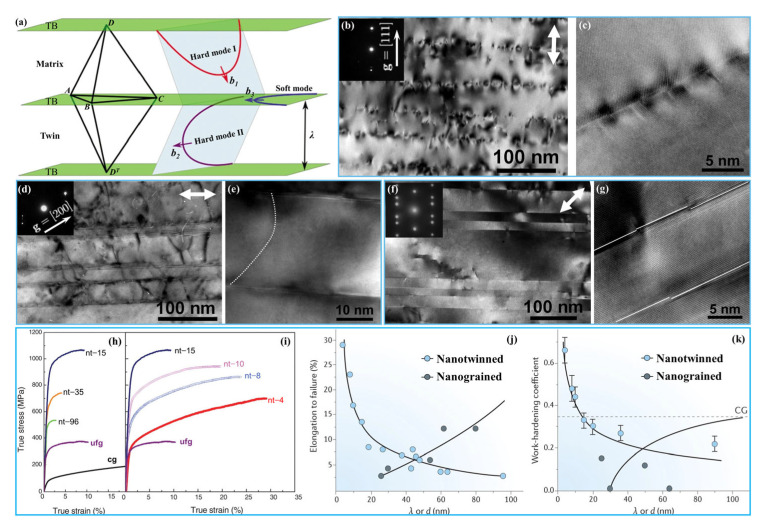
(**a**) Schematics showing the relative orientations between a double Thompson tetrahedra and twin lamellae, where b_1_, b_2_, and b_3_ represent the slip direction of different modes [111]; TEM image and HRTEM images of NT Cu in different loading directions, where the arrow represents the loading direction [110]: (**b**,**c**) 90° compression with a strain of ~5%; (**d**,**e**) 0° compression with a strain of ~6% (the white dash lines represent threading dislocation); (**f**,**g**) 45° compression with a strain of ~7% (the white dash lines represent Shockley partial dislocation). The tensile true stress–strain curves of NT Cu with different λ (cg and ufg refer to the samples with coarse grain (10 μm) and ultrafine grain (500 nm), respectively) varying (**h**) from 15 nm to 96 nm and (**i**) from 4 nm to 15 nm [60]; (**j**) the relationship between λ or d and elongation to failure, and (**k**) the relationship between λ or d and work-hardening coefficient (the dashed line represent the coarse-grained counterpart) [94].

TBs have different impeding effects on both parallel and inclined slip systems during plastic deformation [94,110]. The interactions between dislocations and twins can be divided into three types: hard mode I, hard mode II, and soft mode, according to the relative orientations between slip plane, slip direction, and TBs, as shown by Figure 5a [47,80,94,110,111,112].

In hard mode I, both the slip plane and slip direction are inclined to the TBs. The incident dislocation slides along the {111} <110> slip system inclined to the TBs and accumulates along the TBs, and then cuts across the TBs and leaves numerous residual dislocations. The strengthening of this mode is similar to GB strengthening and also obeys the Hall–Petch relationship, and the slip resistance is proportional to λ^−1/2^. For hard mode II, the slip plane is inclined to TBs and the slip direction is parallel to the TBs. The threading dislocations inside the twin lamellae preferentially nucleate from the intersections between TBs and GBs, then slip in the twin lamellae, and the slip resistance is proportional to λ^−1^. In the case of soft mode, both the slip plane and the slip direction are parallel to the TBs. The Shockley partial dislocations nucleate from the intersections between the TBs and GBs, then slip along the twin planes, leading the TBs to migrate. The slip is not hindered or restricted by the TBs, and the slip resistance is thereby quite low, so the resulting strengthening role decreases in a sequence of hard mode I, hard mode II, and soft mode.

In the compression experiment of an NT Cu sample with λ of ~1.5 nm synthesized by direct current electrodeposition, the YS was 598 ± 31 MPa and 463 ± 16 MPa, respectively, when the compression axis was oriented at 90° and 0° with respect to TBs, but it only exhibited a minor work-hardening ability. In contrast, the compression test with a loading axis oriented at 45° to TBs showed a lower YS of 230 ± 19 MPa, but revealed a large work hardening after yielding [110,111]. This change of loading direction affected the interactions between dislocations and TBs, leading to different deformation mechanisms and, thus, the different YS and work-hardening behavior of the NT Cu.

As shown by Figure 5b,c, when the slip system glides in hard mode I, i.e., under the condition of the loading axis oriented at 90° with respect to TBs, numerous dislocation debris are accumulated along the TBs. These dislocations may be incident dislocations piled against the TBs, Shockley partial dislocations, or Lomer–Cottrell dislocation locks originating from the reactions between dislocations and TBs [110]. The microstructure compressed at 0° revealed much fewer dislocation pile-ups and slips across TBs, and most of the dislocations are threading dislocations (Figure 5d,e), which are confined in the twins or matrix lamellae, and some threading segments are spanned individual twin or matrix lamella with misfit segments lying in the TBs, corresponding to the status of hard mode II. After 45° compression, the TBs are clear and straight and there are no numerous dislocation pile-ups (Figure 5f). Many Shockley partial dislocations with the Burgers vector parallel to the twin plane are clearly seen in Figure 5g, manifested as the steps on TBs. The motion of partial dislocations and migration of TBs lead to the twin lamellae being thickened or narrowed [47]. The dislocation slip is not hindered by the TBs, and thus the strengthening effect is the weakest. In this case, the slip behavior belonged to the soft mode [84].

(2) Effect of λ on strengthening and toughening mechanisms

The λ represents the density of the nanotwins and is the most important factor that affects the mechanical properties of NT metals [60]. Generally, the lower the SFE, the higher the nanotwins’ density, i.e., the smaller the λ. The λ has a significant effect on the deformation mechanism of NT metals and determines the free path of dislocations motion. The smaller the λ (larger than a critical size), the smaller the free path of dislocations, and the larger the blocking effects of TBs to dislocations [84], and, thus, the higher the strength.

When λ is very large (>200 nm), the blocking effect of TBs on dislocations is relatively small. In this case, the plasticity is mainly governed by the conventional interaction of dislocation–dislocation, and the deformation behavior is similar to that of coarse-grained metals. As λ is reduced below ~100 nm, the interactions between dislocations and TBs will dominate the plastic deformation. In this case, the effect of TBs on blocking dislocation motion is similar to that of conventional high-angle GBs, and the difference is that TBs can react with dislocations and provide ample space for dislocation storage [110]. The resulting dislocation density is one to two orders of magnitude higher than the saturated one in traditional polycrystalline metals, which greatly improves the work-hardening capability and, thus, the plasticity [47,60,109]. Therefore, the interactions between dislocations and twins are the main factor for achieving both strengthening and toughening when λ is as small as nanometer size.

As the λ continues to decrease, the number of dislocations piled up against the TBs are gradually decreased, and the stress required for dislocations to cut across the TBs is also increased, i.e., the hard mode I is operated, leading to more significant strain hardening [103]. The research also showed that the YS and ductility increased with decreasing λ in NT Cu, and reached a maximum value of ~1 GPa as the λ decreased to a critical size (~15 nm), and simultaneously a uniform elongation of 13% was achieved (Figure 5h). At this critical condition, the strengthening mechanism of the equiaxed NT Cu will change from the Hall–Petch strengthening, in which dislocations pile up against the TBs, to the softening mechanism, where the pre-existing Shockley partial dislocations move parallel to the TBs (i.e., the soft mode) [60], so further decrease of λ reveals a monotonic decrease in strength (Figure 5i) but significant increases in elongation to failure and work-hardening coefficient (Figure 5j,k). In particular, the work-hardening coefficient can exceed the upper limit of the corresponding coarse-grained Cu when λ is decreased to a few nanometers [94]. The ultrahigh work-hardening effect came from the fact that the numerous TBs can effectively absorb high density of dislocations. Therefore, as the λ falls below the critical size, the strength of the NT metals gradually decreases, while the plasticity monotonically increases.

Besides the effects from the above experiments, Li and coworkers [113] used molecular dynamics (MD) simulations to attempt to clarify the effect of λ on mechanical properties of NT Cu with different values of λ, and the results also indicated that the strength increased as the λ decreased, reaching a maximum at a critical λ value of ~1.25 nm, and then decreased, but the elongation increased monotonically. This further demonstrates that the most excellent comprehensive mechanical properties can be obtained at the critical λ value. As the λ exceeds the critical value, the plastic deformation mechanism will change from the interactions between dislocations and TBs to partial dislocation nucleation and movement at GB–TB intersections. In addition, the simulation results also showed that the critical λ depended on the grain size; the smaller the grain size, the smaller the critical λ, and the higher the maximum strength, while the ductility increases continuously.

In addition to the standpoint that the interactions between dislocations and TBs could bring about the strengthening and toughening, Guo et al. [114] found that full dislocations were “repelled” by TBs on the free surface of NT Pt films used in in situ tensile tests, i.e., the generated repulsive force between the TBs and dislocations was another reason for the high strength of NT structures. However, the transition of deformation mechanism with λ was observed in various metals such as NT Cu, Ag, and Pt, but the softening effect with decrease of λ was not observed in NT Ni [108], suggesting that the softening effect from λ did not apply to all NT metals. Therefore, the mechanisms of strengthening and toughening in NT metals, especially the associated essence between λ and deformation mechanism, need to be further clarified.

In summary, nanotwins are classified into deformation twins and growth twins. Sufficient dislocations and high local stress during plastic deformation can drive the nucleation and growth of deformation nanotwins, while the stress relaxation during deposition may be the main reason for forming growth nanotwins. The main preparation techniques for nanotwins include DPD, PVD, and electrodeposition methods. Although the DPD method is capable of preparing millimeter-scale samples, the microstructure is a mixture of nanograins, dislocations, and nanotwins, while that achieved by the two deposition methods is always an ideal clean high-density NT structure, but the thickness of the resulting sample is generally in the micrometer-scale. The NT metals usually have excellent mechanical properties due to the unique interactions between dislocations and nanotwins. However, the strengthening and toughening mechanisms of NT structures are not consistent, such as the interactions and the repulsive force between dislocations and TBs. Moreover, the influence mechanism of λ, especially the critical λ, on the transition of deformation mechanism was also unclear. Due to the difficulty in fabricating NT metals with a specific λ value, there are a few experimental studies on the more detailed effect of λ on the strengthening and toughening mechanisms, especially the effect below the critical λ. All of these should be further clarified. In particular, although it has been found that the lower the SFE, the smaller the λ, how to quantify the relationship between SFE and λ is also an area of interest.

### 3.3. HNT Structure

#### 3.3.1. The Preparation of the HNT Structure

To further increase the numbers of TB in different directions and provide more pathways for dislocation motion (such as glide and cross-slip), and thus improve the mechanical properties, the HNT structure has been proposed in recent years [115]. Similar to the NT structure, the hierarchical nanotwins also include two kinds, growth nanotwins and deformation nanotwins, according to their formation mechanisms. Different from the NT structure, the growth of the HNT structure has been only prepared in a few metals, such Ni–Mn–Ga shape memory alloy [116] and single-crystal Ni alloy [117]. The most common preparation method is still the severe plastic deformation, such as equal channel angular pressing, surface mechanical treatment, stretching after preloading, and so on, and the achieved products are the deformation-induced HNT structures [118,119,120].

**Figure 6:**
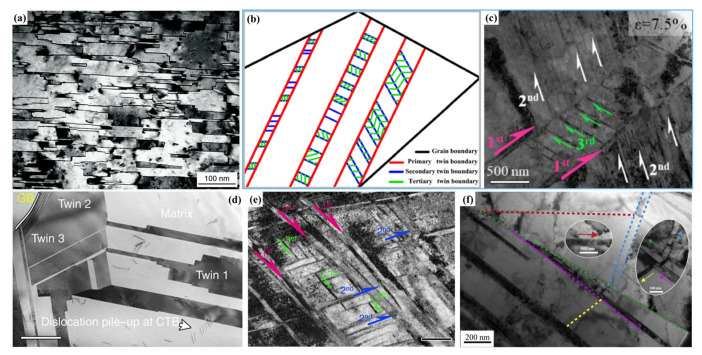
(**a**) TEM micrograph of the high-density stepped nanotwins in single-crystal Ni [117]; (**b**) a schematic of the HNT structure in Ti–4Mo–3Cr–1Fe alloy [121]; (**c**) TEM image of three-orders HNT structure in CoCrFeMnNi alloy [122]; (**d**) the hierarchical twinning architecture in a grain of CrCoNi alloy [123]; (**e**) the three-orders HNT structure in TWIP steel [120]; (**f**) TEM image of five-orders HNT structures (the primary, secondary, tertiary, quaternary and quinary TBs are marked sequentially in red, blue, pink, yellow and green), and insets reveal the details of the corresponding ordered twins in the same colored arrows [124].

Li et al. [117] integrated sputtering and electrodeposition methods to prepare the HNT structure in single-crystal Ni, and the resulting structure contained mutually parallel coherent TBs interrupted by multiple vertical incoherent TB segments, showing a two-orders HNT structure with an average coherent twin lamellae spacing of ~22 nm (Figure 6a). The sample had an ultrahigh strength of ~2 GPa and significant deformation ability of 19% in in situ compression.

Similar to the NT structure, the SFE is also the decisive factor for the formation of HNT structure, and lower SFE is helpful for forming higher-order twin structure [107,118], i.e., HNT structures can be generated in low-SFE metals such as Mg alloy [125], Ti alloy [121], high-entropy alloy [126], twinning-induced plasticity (TWIP) steel [120], etc. Fu et al. [125] obtained two-order HNT structure in Mg–8 wt.% Li alloy by one-step ultrahigh-pressure and high-temperature procedure, i.e., the sample was treated for 30 min at temperature from room temperature to 1200 °C under high pressure of 6 GPa, then quenched to room temperature, in which there was a continuous staggered twin network. The sample had a high tensile YS of ~249 MPa (~4.05 times that of the as-cast counterpart) and a good elongation of ~23.6%. In order to further improve the YS (~145 MPa) of a two-order HNT Ti–12Mo alloy [127], a highly stable Ti–4Mo–3Cr–1Fe HNT alloy was designed and prepared through multipass forging. The microstructure evolution during the tensile process indicated that twins within the grains gradually changed from primary to tertiary twins, and the volume fraction of twins increased while the twin spacing decreased. The tertiary lamellar twins had a thickness of ~15 nm and were uniformly distributed, as shown by the schematic image in Figure 6b [121]. The tensile YS, UTS, and elongation of the sample were 870 MPa, 1092 MPa, and 41%, respectively, improved by 93%, ~11%, and ~8%, respectively, compared with those of the HNT Ti–12Mo alloy.

Inspired by the idea of forming the HNT structure through combining different types of twins, Chung et al. [126] first prepared primary lamellar annealed nanotwins in an Fe_22_Co_22_Ni_20_Cr_22_Mn_14_ high-entropy alloy by annealing, and then induced secondary and tertiary streak-like deformation nanotwins by subsequent high strain rate (9 × 10^−3^ s^−1^) compression at cryogenic temperature (−150 °C). The obtained HNT structure significantly improved the work-hardening ability, had an excellent compression strength of ~3.3 GPa, and a high ductility of ~31.9%. This result implied that combining different types of twins (annealing and deformation twins) through controlling the preparation process may be a novel approach for designing a high-order HNT structure with high performance. In order to reduce the SFE of equiatomic CoCrFeMnNi high-entropy alloy, and thus encourage the formation of deformation twins, An et al. [122] prepared a three-order HNT structure in a nonequiatomic Co_21.5_Cr_21.5_Fe_21.5_Mn_21.5_Ni_14_ alloy by further reducing the SFE (reduced from 25 mJ/m^2^ to 7.7 mJ/m^2^) through adjusting the alloy composition and then utilized the shot peening method to realize severe deformation (Figure 6c). The resulting alloy exhibited a high tensile YS of ~750 MPa and a good uniform elongation of ~27.5%; the YS was ~4 times higher than of the cast alloy, and the elongation was equivalent to that of the cast counterpart. Similarly, a three-order HNT structure was also fabricated in a CrCoNi medium-entropy alloy by cold forging and subsequent rolling at room temperature. The in situ TEM tensile test revealed that three twinning systems were activated within individual grains during deformation, forming a three-dimensional (3D) twin network structure, in which multiple twins with different thicknesses formed and intersected each other (Figure 6d), achieving a high UTS of ~1 GPa and an excellent uniform elongation of ~60% [123,128].

In addition to high/medium-entropy alloys, Wei and coworkers [120] prepared a three-order HNT structure in a TWIP steel by using pretorsion and subsequent tensile deformation (Figure 6e). The tensile testing indicated that the YS of the sample can be doubled with no reduction in ductility, which further proved the advantage of the HNT structure in maintaining a good ductility and a high strength. In view of the superiority of the HNT structure in TWIP steel [120], an HNT structure with five orders was successfully fabricated in pure Ag via combining the uniaxial preloading and surface mechanical attrition treatment (SMAT) technique (Figure 6f) [124]. The YS reached ~145 MPa, ~3.5 times higher than that of the annealed Ag, and the ductility of ~35% was only slightly sacrificed. In particular, both the YS and ductility of the five-orders HNT Ag were better than those of the two-orders HNT Ag (~125 MPa and ~24%) [68].

From the above discussion, it can be concluded that achieving the HNT structure is a promising way to enhance the comprehensive mechanical properties of metals. Similar to the NT structure, the preparation of the HNT structure is still mainly based on deformation-induced nanotwins, and the growth of the HNT structure can only be formed in a few metals. In addition, there are few HNT structures above five orders, and almost all the metals mentioned above have a mixed hierarchical nanostructure composed of dislocations, nanograins and nanotwins, similar to those prepared by the DPD approach. Therefore, further research is still needed for developing fabrication technology of clean and high-order HNT structure.

#### 3.3.2. Strengthening and Toughening Mechanisms

The mechanical properties of the HNT structure have been comprehensively investigated in terms of theoretical modeling and experimental examination [115,129,130,131]. As mentioned above, λ is the most important parameter for charactering NT structures and has an important effect on the mechanical properties and deformation mechanisms; similarly, it also has an important role in the HNT structure.

**Figure 7:**
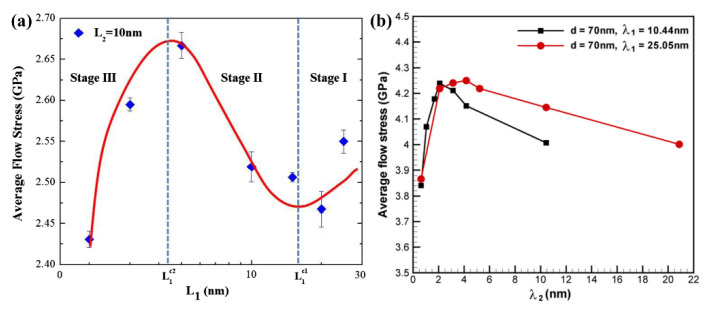
(**a**) The relationship between average flow stress and primary twin spacing λ_1_ in the HNT Cu [132], and (**b**) the relationship between average flow stress and secondary twin spacing λ_2_ in two-order HNT Cu using MD simulation [133].

However, since accurately controlling λ in an HNT structure is quite difficult, a significant proportion of the existing studies have used theoretical calculations and simulations to clarify the relationship between λ and mechanical properties [132,133]. Sun et al. [132] studied the deformation mechanisms of the HNT Cu with different primary twin spacings (λ_1_), but the same grain size, d, and the same secondary twin spacing (λ_2_) by MD simulations. The results indicated that the deformation mechanisms were closely related to two critical values of λ_1_ ($L_{1}^{c1}$ and $L_{1}^{c2}$), which corresponded to two softening stages and one strengthening stage, as shown by Figure 7a. When λ_1_ was larger than the first critical primary twin spacing $L_{1}^{c1}$, the deformation changed from full dislocation-dominated plastic deformation to partial dislocation-dominated, which caused a softening (stage I). However, when λ_1_ was decreased to a value between $L_{1}^{c1}$ and $L_{1}^{c2}$, the strength increased with decreasing λ_1_ due to the transition from partial dislocation to dislocation blockage (stage II). As λ_1_ was further reduced below $L_{1}^{c2}$, the strength decreased again because of the deformation mechanism of partial dislocation motion-induced TBs migration and detwinning (stage III).

In addition to the effect of λ_1_, the influence of λ_2_ on the strength was also explored. Yuan et al. [133] studied the effect of both λ_1_ and λ_2_ on the strength of the HNT Cu by MD simulations, and found that at the conditions of same grain size *d* and λ_1_, the average flow stress first increased as λ_2_ decreased, reached a maximum at a critical λ_2_, and then decreased as λ_2_ further decreased (Figure 7b). In addition, a larger λ_1_ resulted in a larger critical λ_2_. As λ_2_ decreased over the critical value, the original Hall–Petch strengthening mechanism in which partial dislocations that are emitted from GBs and TBs and cross other GBs or TBs would transform into a softening mechanism caused by detwinning of secondary twins and migration of primary twins, resulting in a maximum strength at this critical λ_2_ [67,133]. Li et al. [134] developed a theoretical model by MD simulation of the HNT Cu and found that there was a critical λ_1_ value that made the strength reach a maximum value, and it was proportional to grain size. The peak strength could reach a high level when all the high-order twin thicknesses were larger than λ_1_. In addition, it was proposed that to obtain the best mechanical properties was to achieve a two-order HNT structure, in which λ_1_ was maintained at its critical value while λ_2_ was enlarged as much as possible.

In summary, in addition to conventional TB strengthening, the HNT structure can serve as pathways for dislocation glide along, and cross-slip between, intersecting TB–matrix interfaces, and thus is able to considerably enhance strength without sacrificing ductility, achieving higher mechanical properties than the NT structure. Similar to the status of the NT structure, there is also a critical λ value for each-order twin in the HNT structure, showing different deformation mechanisms, and thus different mechanical properties on both sides of them. The deformation mechanisms and related strengthening and toughening mechanisms of the HNT structure are very complicated due to the interactions between different-order twins, which mainly have been investigated by numerical simulation, but needed to be further verified by experiments.

### 3.4. Summary and Prospect

The NT and HNT structures are a new way to simultaneously strengthen and toughen nanostructured metals. The DPD is the most commonly used method for preparing NT and HNT structures, but the resulting structures are always mixtures of nanograins, dislocations, and nanotwins. In contrast, deposition methods such as PVD and electrodeposition can achieve clean structure with unique nanotwins and ease of controlling λ precisely, but can only be formed in a few metals. In addition, the DPD method can obtain metals of millimeter-scale, but the deposition method can only obtain metals of micron-scale thickness, so the DPD method is more promising from the perspective of engineering applications, while the deposition method is suitable for the preparation of metals for theoretical studies. Since the HNT structure is basically prepared only by the SPD method, less metal can be obtained with the HNT structure than with the NT structure, regardless of the method. Consequently, for preparation techniques, especially the DPD method, new deformation technology needs to be developed to obtain thicker (larger size) and cleaner NT and HNT structures.

The level of SFE is the decisive factor for the formation of NT and HNT structures, while the interactions between TBs and dislocations are the source of their excellent mechanical properties. The lower the SFE, the easier the twinning deformation and the higher the nanotwins’ density (i.e., the smaller the λ). As the λ decreases (greater than a critical value), the number of dislocations piling up against the TBs gradually decreases, and the applied stress required for dislocations to cut through TBs increases, which contributes to high strength and significant strain hardening. In addition to the above-mentioned reason, it has also been proposed that the repulsive force between the TBs and dislocations is another reason for high strength. The TBs can absorb and store dislocations to withstand larger plastic deformation, thus achieving a high toughness. Compared with the NT structure, the unique 3D nanotwin network of the HNT structure can offer the required pathways for multiple movement modes of dislocations (such as slip and cross-slip), resulting in a more homogeneous distribution of dislocation activity in three dimensions and further improving the plastic deformation ability. Based on the above discussion, it is found that there are still some issues that need further studied. How to reduce the SFE of more metals by alloying, so as to introduce the NT or HNT structure to obtain fascinating mechanical properties, and how to quantify the relationship between SFE and the λ will be the focus of the next research. In addition, research on the limitations of nanotwin orders and the sole strengthening and toughening mechanisms of the HNT structure is still also a very challenging topic.

Finally, combining structural design, computational simulation, preparation technology, and other perspectives to develop the NT/HNT metals with higher strength and toughness will be the focus of structural metals research.

## 4. Gradient Nanostructure

In 1984, M. Niino et al. [135] proposed the concept of gradient structure in ceramic/metal coating, in which both the composition and the structure gradually change over the volume, resulting in corresponding changes in the properties of the materials. Latterly, it was found that the heterogeneity and multiscale of the microstructure constituents in gradient distributions could provide extra strain hardening and suppress strain localization, and thus improve the mechanical properties of metals. Reasonably, the gradient structure was introduced into nanostructured metals in order to further improve their mechanical properties, especially to overcome their low ductility [136].

Gradient-nanostructured metals typically have a gradient in the interior microstructure, such as grain size, twin thickness, and/or lamellar thickness, over a characteristic length scale from the surface to the interior, ranging from nano-level to sub-micrometer, or even to millimeter, and show a spatial gradient distribution [26,27,49,136,137,138]. The essence of a gradient nanostructure is that the density of interfaces such as GBs (TBs) varies in a gradient form without obvious interfaces, forming gradient changes in physical properties, such as wear resistance and corrosion resistance [139].

**Figure 8:**
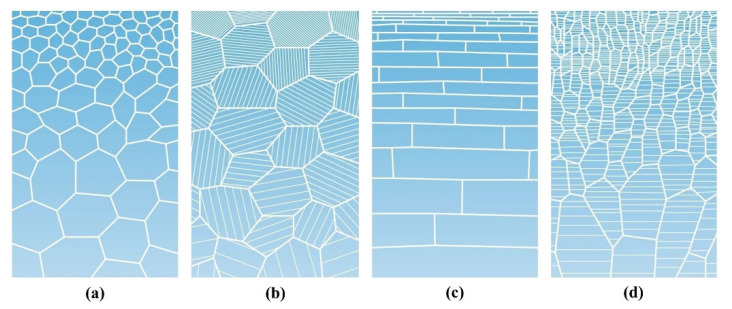
The classification of gradient nanostructure [27]: (**a**) grain size gradient; (**b**) twin thickness gradient; (**c**) lamellar thickness gradient; (**d**) columnar size gradient.

Lu et al. [26] summarized four basic configurations of gradient-nanostructured metals with the same chemical and phase composition: (1) gradient-nanograined (GNG) structure: the structure unit is equiaxed (or approximately equiaxed) grains, and the grain size changes in a gradient manner from tens of nanometers to microns from the surface to the interior of a metal (Figure 8a); (2) twin thickness gradient structure: the grain size is uniform and there are nanotwins inside the grains, but the twin thickness changes from nano-level to near-macro-scale gradually (Figure 8b); (3) lamellar thickness gradient structure: the structure unit is composed of two-dimensional lamellar grains whose thicknesses vary gradually from nanometer to micrometer (Figure 8c); (4) columnar size gradient structure: the structure unit consists of one-dimensional columnar grains, and the diameter of the columnar grains changes gradually from nanometer to near-macro-scale (Figure 8d).

In general, according to the above types of gradient nanostructures, they can be summarized into two types: one is GNG structure, and its structure unit is equiaxed, lamellar, or columnar grain, in which the grain size or lamellar thickness exhibits a gradient change [136,140,141]. The other is gradient-nanotwinned (GNT) structure, in which the twin lamellar thickness shows a gradient change [142,143]. At present, the gradient nanostructure is mainly prepared by gradient plastic deformation and physical or chemical deposition.

### 4.1. Gradient Plastic Deformation

The basic procedure of the gradient plastic deformation method is to produce severe plastic deformation on a sample surface through multiple or repeated plastic deformation. The resulting deformation amount, rate, and temperature from the surface to the interior all exhibit gradient changes, thereby a gradient nanostructure forms on the metal surface. The involved deformation methods include SMAT [144,145,146,147], surface mechanical grinding treatment (SMGT) [136,148], surface mechanical rolling technique (SMRT) [149,150,151,152], and other plastic deformation technologies (such as laser shock peening, shot peening, etc.) [153,154].

(1) GNG structure

**Figure 9:**
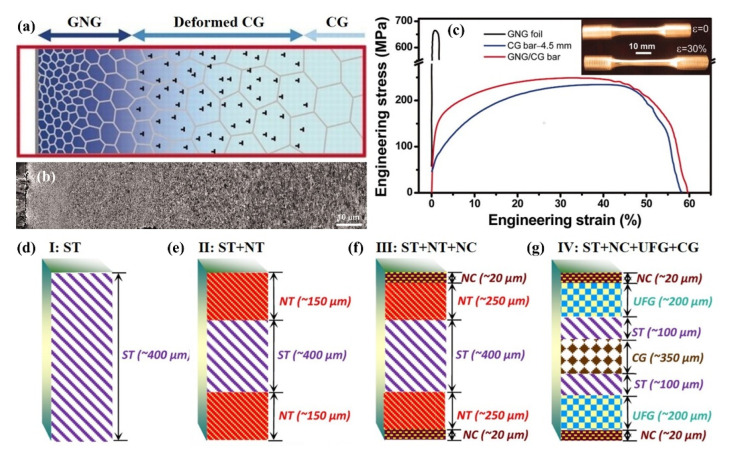
(**a**) Schematic of the cross-sectional microstructure of GNG Cu (CG refers to the coarse grain), where the solid symbols represent dislocation tangles or dislocation cell [136]; (**b**) a cross-sectional SEM image of an SMGT Cu sample [136]; (**c**) the tensile engineering stress–strain curves for the CG Cu, GNG Cu, and a freestanding GNG foil sample, and inset shows the tensile GNG Cu sample before and after tension [136]; schematic images of four types of gradient-structured 304 SS [143] (ST, NT, NC, UFG), and CG refers to sub-micrometer twins, nanotwins, nanocrystalline, ultrafine grains, and coarse grains, respectively); (**d**) ST; (**e**) ST + NT; (**f**) ST + NT + NC; (**g**) ST + NC + UFG + CG.

In 2011, Lu et al. [136] prepared gradient-nanograined Cu film on a coarse-grained Cu substrate by SMGT technique for the first time, and the grain size varied from ~20 nm to ~300 nm in a range of ~150 μm from the surface (Figure 9a,b). The tensile YS of the sample was 129 MPa, ~2 times the YS of the coarse-grained Cu, and the uniform elongation was the same as that of the coarse-grained counterpart, showing a good synergy of strength–ductility (Figure 9c). Similarly, Wu et al. [144] produced a ~120 μm thick GNG structured layer by SMAT in an interstitial free (IF) steel, and the mean grain size from surface to center gradually increased from ~96 nm to ~35 μm. The elongation of the sample was basically the same as that of the coarse-grained one, but the YS was ~2.6 times higher than the latter.

In order to broaden the utilization of gradient structure, Chen et al. refined the grains of the topmost layer of AZ31B Mg alloy to ~21 nm, while the grains in the center remained ~30 μm through the SMAT process, and the thickness of the whole gradient-structured layer was ~140 μm. The tensile YS of the resulting sample increased from ~147 MPa of the as-cast sample to ~249 MPa, and the uniform elongation of ~9.3% was only reduced by 6.1% [145]. In addition to single-sided GNG structure, a double-sided GNG layer structure was prepared successfully using SMAT on a WE43 Mg alloy, and the grain size and dislocation density of the ~300 μm thick GNG layer varied from ~38.2 nm and 4.5 × 10^14^ m^−^^2^ of the top layer to ~230 nm and 0.1 × 10^14^ m^−2^ of the center, respectively [147]. This GNG structure possessed a high YS of ~435 MPa and a reasonable ductility of ~11%, increased by ~172% and ~47%, respectively, compared with the those of the as-cast sample. That is, the double-sided GNG structure was twice as strong as the single-sided GNG structure while maintaining good elongation. It was proposed that the volume fraction of coarse grains determines the ductility while that of fine grains determines the strength. There is a critical value for the volume fraction of coarse grains or fine grains, at which the best strength–ductility synergy can be achieved. Similarly, Wu et al. [144] also suggested that there should be an optimal gradient structure layer thickness that led to the maximum strength in the aforementioned GNG IF steel.

In addition to the equiaxed GNG structure, the lamellar GNG structure has also been developed. The formation of the lamellar structure is reasonably attributed to the large shear deformation with very high rates in the top surface layer of the sample, which is distinct from the conventional SPD processes such as cold rolling. Liu et al. [155] applied a very-high-rate shear deformation in the top surface layer of a pure bulk Ni sample, where a nanometer laminated structure was induced. With the depth increased, the microstructures in the sequence showed equiaxed nanograins with an average size of ~11 nm in the range of 10 μm, lamellar grains with average thickness of 20 nm across the depth span of 10 μm to ~50 μm, and a transition layer from the lamellar structure to the ultrafine grain with an average size of 70 nm in a depth span of 50 μm to ~80 μm. The sample showed an ultrahigh hardness of 6.4 GPa, much higher than that of nanograined Ni (~3.4 GPa). After that, Wang et al. [140] fabricated a lamellar GNG structure ~30 μm thick on an AISI 420 SS by laser shock peening. At the topmost layer, fine equiaxed grains in a size range of 14–38 nm were observed; as the depth varied from 5 μm to 30 μm, the thickness of martensite lamellae increased from ~200 nm to ~650 nm, and the resulting sample had a UTS of ~535 MPa and a total elongation of ~30.6%, which was 24.3% and 47.8% higher than the coarse-grained counterpart, respectively. A gradient lamellar nanostructure ~300 μm thick was also prepared on an IF steel through SMRT; as the depth increased, the microstructure was in the order of nano-lamellar grains with a mean thickness of ~80 nm at the depth of 10 μm, ultrafine-lamellar grains where the mean lamellar thickness reached ~160 nm at the depth of 10–100 μm, and deformed coarse-lamellar grains with grain sizes of hundreds of micrometers in the center. The YS, UTS, and failure to elongation increased from 91 MPa, 220 MPa, and 46% for the coarse-grained sample to 127 MPa, 248 MPa, and 52% for the GNG one, respectively [156].

From the above discussion, it is clear that the mechanical properties of the double-sided gradient structure are better than those of the single-sided one. In addition, for the lamellar GNG structure, a higher strain rate of deformation is beneficial to form lamellar structures with larger volume fractions and smaller grain sizes, so it has a greater enhancement of mechanical properties than equiaxed GNG structures. The higher the strain rate of deformation, the easier it is to form a lamellar structure with a larger volume fraction, and the smaller its grain size, and therefore it tends to have a greater enhancement of mechanical properties than equiaxed GNG structures.

(2) GNT structure

For metals with high SFE, only the GNG structure is formed during gradient plastic deformation, while for low-SFE metals, the GNT structure or other gradients, such as dislocation density gradient, are also formed at the same time, in addition to the GNG structure, i.e., the resulting GNT structure accompanied by a dual or multiple gradients of GNG structure.

Wang et al. [157] prepared an Fe–25Mn steel sample with GNT structure via SMGT process, and the grain size increased from ~40 nm to ~50 μm and the average λ increased from ~11.2 nm to ~65.7 nm from the surface to the center, respectively, showing dual gradient distribution of grain size and twin density. In addition, it was found that combining hot swaging with turning technology can easily fabricate a GNT CrCoNi medium-entropy alloy because of its relatively low SFE [158]. The grain size in the 150 μm thick GNT layer increased from ~25 nm in the topmost surface to ~17 μm in the center, and the average λ gradually increased from ~8 nm in the topmost surface to ~130 nm at depth of ~80 μm. This GNT structure exhibited a significant increase in YS from 450 MPa, of the original casting sample, to 1100 MPa, while maintaining a good total elongation of 27%. Compared with the coarse-grained counterpart, the YS of the GNT steel was increased by ~75%, but the ductility was only sacrificed by ~19%. Further, Cheng et al. [142] fabricated a 310 μm thick gradient structure composed of two gradient surface layers that sandwich a coarse-grained center in high-manganese steel by rotationally accelerated shot peening. The lamellar thickness increased from ~20 nm to ~100 nm as the depth increased from 20 μm to 80 μm, and high-density nanotwins with lamellar thickness of several nanometers were embedded in the grains. Further increasing the depth, the lamellar thickness coarsened to ~400 nm. That is, the sample showed a dual gradient distribution of nano-laminated grains and nanotwins. The YS and UTS of the GNT sample increased, from 249 MPa and 786 MPa of the coarse-grained counterpart, to 392 MPa and 842 MPa, while the elongation remained comparable. 

For multigradient microstructure, Chen et al. [143] fabricated four types of gradient microstructure in 304 SS that were composed of sub-micrometer twins, nanotwins, nanocrystalline, ultrafine grains, or coarse grains by SMAT process (Figure 9d–f). Compared with the coarse-grained structure, the four gradient structures all had higher mechanical properties. The sample with sub-micrometer twins and nanotwins exhibited better performance of work hardening, which was beneficial for delaying necking and maintaining high plasticity, and the resulting ductility was ~50% higher than that of the coarse-grained sample. It is expected that the sub-micrometer twin-strengthened metals may have broader significance in engineering applications compared with nanotwins because they are easier to achieve.

Gradient plastic deformation is a simple and efficient preparation method for preparing GNT structures, but the depth (micrometer-scale) and the volume fraction (20–25%) of the gradient structure layer in the obtained sample are very limited. Similar to DPD and SPD methods, the resulting microstructure from gradient plastic deformation is not “clean” and is only suitable for metals with good plasticity. GNT structures with grain size and twin thickness gradient are often realized in low-SFE metals to further obtain high strength and high toughness.

### 4.2. Physical/Chemical Deposition Method

To increase the volume fraction of gradient structure in the sample, the physical/chemical deposition methods were developed, which mainly include electrodeposition and magnetron sputtering, and a precisely controllable gradient structure can be achieved by controlling the physical or chemical deposition kinetic process through adjusting the parameters such as temperature, current density, additive types, contents, etc. [26]. In addition, the resulting gradient metals usually have a “clean” structure with fewer defects and high density of twins, and, thus, generally have good mechanical properties.

(1) GNG structure

A GNG structured pure Ni foil with grain size varying from ~29 nm to ~4 μm was prepared using the electrodeposition method, with grain sizes varying from nanometer-scale to micrometer-scale [159]. The sample had a YS of ~460 MPa and an elongation of ~8.9%, and the YS was increased by 21% while the elongation was even higher than the 7.5% of the coarse-grained counterpart. A symmetric double-sided GNG Ni–P alloy with a total thickness of 600 μm was prepared by double-sided electrodeposition on a coarse-grained Ni substrate with a thickness of 100 μm [160]. The grain size varied from ~7 μm in the center to ~10 nm in the topmost surface, and the total volume fraction of nanograins was 35%. The YS and UTS significantly increased from ~100 MPa and ~218 MPa of the coarse-grained Ni to ~539 MPa and ~662 MPa, respectively, while the uniform elongation was sacrificed from ~18% to ~4.9%.

**Figure 10:**
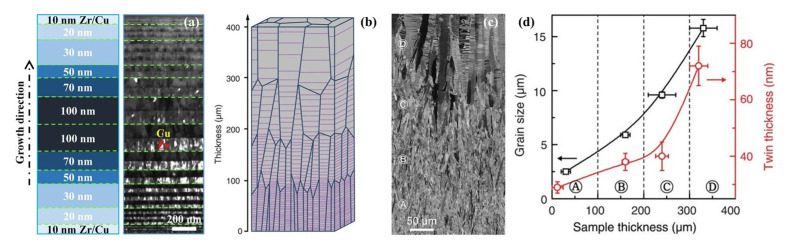
(**a**) Schematic and TEM image of the gradient-nanolayered Cu/Zr metal [162]; (**b**) schematics and SEM observation (**c**) of GNT Cu composed of homogeneous NT components Ⓐ, Ⓑ, Ⓒ and Ⓓ [28]; (**d**) distribution of grain size and twin thickness along the sample thickness (the directions of the arrows represent the corresponding vertical axis, respectively) [28].

Similarly, a gradient nanolayer Ni with a thickness of ~2.3 mm was fabricated through electrodeposition by Gu et al. [161], which consisted of alternating layers of ultrafine (~300 nm) and nano (~18 nm) grains. The sample exhibited an ultrahigh UTS of ~1150 MPa and a good elongation to failure of ~6.6%, a ~28% increase in strength but only ~2% reduction in ductility compared the ultrafine-grained Ni. Moreover, it was also found that the mechanical properties of the double-sided GNG structure are greater than those of the single-sided GNG structure. Inspired by the GNG Cu [136], Li et al. [162] also prepared a gradient-nanolayered metal composed of the alternate soft Cu and hard Zr nanolayers by magnetron sputtering; the layer thicknesses of the two metals gradually increased from 10 nm in the two side-surfaces to 100 nm in the center, forming a symmetrical thickness-gradient distribution of the two metal layers (Figure 10a), i.e., a double-sided symmetric gradient structure, contributing a uniform deformation of ~60% and a yield stress of ~1.9 GPa in micropillar compression. It can be seen that the mechanical properties of the nanolayered GNG structure tend to be higher than those of the equiaxed GNG structure.

(2) GNT structure

In 2018, Cheng et al. [28] prepared a GNT Cu with dual gradient distributions of grain size and twin density by direct-current electrodeposition (Figure 10b,c). The λ increased from 29 nm to 72 nm, the grain size increased from 2.5 μm to 15.8 μm from the top to bottom of the sample (Figure 10d), the sample exhibited a YS of 364 MPa and a UTS of 397 MPa, and, simultaneously, a uniform elongation of 7% was maintained. 

In contrast to GNG structure prepared by gradient plastic deformation, the physical/chemical deposition method can prepare metals with gradient nanostructure from the surface to interior, and can realize precise control of the structure constituents by adjusting the preparation process. Similar to the NT structure prepared by the PVD method, the gradient nanostructures prepared by the physical/chemical deposition method have a higher volume fraction of the gradient structure and can obtain a “clean” structure, thus exhibiting superior mechanical properties. However, it is also similar to the disadvantages of the PVD method: it has difficulty realizing engineering applications.

### 4.3. Strengthening and Toughening Mechanisms

**Figure 11:**
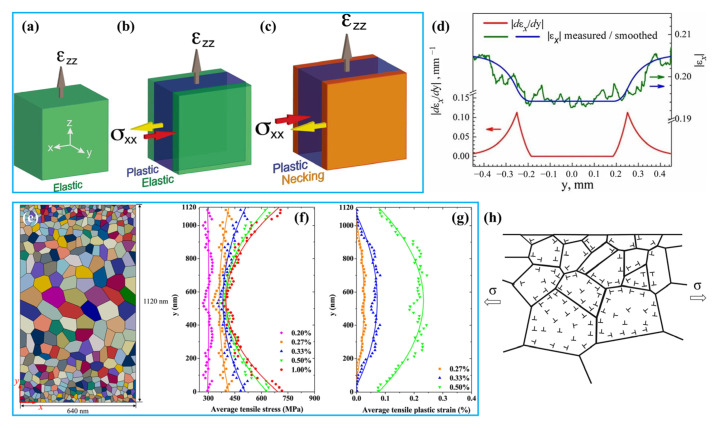
Schematics of the change of stress state during tensile testing of GNG structure [163]. (**a**) Stage I; (**b**) stage II; (**c**) stage III; (**d**) distribution of the lateral strain (ε_x_) and strain gradient (dε_x_/dy) in GNG IF steel [144]; (**e**) crystal plasticity finite-element model of GNG Cu [164]; distribution of (**f**) gradient axial stresses and (**g**) gradient axial strains in the cross-section at various applied strains [164]; (**h**) schematic illustration of GNDs in plastically deformed GNG structure (⟂ represent GNDs near the GBs) [164].

The GNG structure has simultaneously high strength and high ductility, which depend on the gradient distribution of constituents and resultant unique plastic deformation mechanisms [47]. The available investigations have shown that the deformation of the double-sided GNG metals during tensile process can be classified into three stages [51,163]. In stage I, the overall metal deforms elastically at small strain (Figure 11a); in stage II, the central coarse-grained regions start to deform plastically due to lower YS, while the nanograined regions still undergo elastic deformation as the applied strain increases, generating an obvious constraint between the undeformed surface nano-grained region and the deformed central coarse-grained region, and this constraint is continuously moving. This constraint then changes the applied uniaxial stress into biaxial stresses (Figure 11b). As a result, two elastic/plastic interfaces in the symmetrical gradient structure are formed, while only one elastic/plastic interface is generated in the single-sided GNG structure, which all gradually move toward the sample surface as strain increases. In stage III, the whole sample deforms plastically. Both the surfaces of the sample (i.e., the nanograin region) undergo unstable necking first, which causes quick contraction of the sample cross-section (Figure 11c). However, the necking can be constrained by the central stable coarse-grained region, and then a steep strain gradient is generated at the interfaces between the two-side unstable necking regions and the central stable region, which promotes the accumulation of GNDs and the development of back stress near the interfaces, resulting in an increase of strain-hardening ability and, thus, an improvement in ductility.

As mentioned above, the strengthening and toughening mechanisms of the GNG structure are closely related to the plastic strain gradient, GNDs, and the relationship between GNDs and the work-hardening rate during deformation, so both the mechanisms are discussed in detail in terms of these aspects.

#### 4.3.1. Plastic Strain Gradient

Due to the gradient distribution of structure constituents, the deformation amounts of the constituents with different characteristic sizes are not the same under the same stress state, and the deformation amount inside the metal is in a gradient form, i.e., a gradient plastic strain is formed [26,27,160]. The strain gradient leads to the original uniaxial stress state changed into a biaxial stress state, which enhances the storage and interactions of dislocations, generating an additional strain hardening and an increase in strain-hardening rate, and thereby endowing the GNG structure with a superior strength–ductility combination [144].

The strain gradient generated during deformation has been studied by both experiments and simulations. After measuring the height profile on the cross-section normal to the tensile direction of a GNG IF steel at a tensile strain of 0.25, the strain and strain gradient on the cross-section across the thickness direction of the “GNG layer–coarse grains layer–GNG layer” were calculated (Figure 11d). The results indicated that the strains in the two side GNG surfaces were higher than those in the central coarse-grained region (the blue line), and an obvious plastic strain gradient was formed (the red line), revealing a maximum strain gradient near the interfaces between the surface GNG regions and the central coarse-grained region [144]. Recently, Lu et al. [26] also found that the gradient of grain size led to a strain gradient during uniaxial tensile process of GNG Cu, which changed the uniaxial stress state into complex stress state. In addition to the experimental studies, a structural model of GNG Cu during deformation through finite-element modeling was established (Figure 11e) [164]. The result indicated that the tensile stress gradually increased from the center coarse-grained region to the side nanograined region during the tensile process, i.e., also showing a gradient distribution (Figure 11f); correspondingly, there was an inverse gradient distribution of plastic strain (Figure 11g), which was consistent with the experimental results [144]. In addition, Figure 11g also reveals that the strain gradient increased as the tensile deformation proceeded.

Similarly, the plastic strain gradient was also found in other studies on GNG Cu [165], 316 SS [166], 304 SS [167], and IF steel [144]. Therefore, it can be concluded that the strain gradient is a universal phenomenon of GNG metals under stress.

#### 4.3.2. Geometrically Necessary Dislocations

In order to coordinate the strain gradient, GNDs are distributed near the GBs, phase boundaries, or interfaces between the nanograins and coarse grains in the gradient nanostructure (Figure 11h) [26,47,144,168]; the induced GNDs will work-harden and thus strengthen the metals in two ways: (1) strengthening from forest dislocations, in which the short-range interactions between GNDs and mobile dislocations increases the resistance of dislocation slip; (2) strengthening from back stress, which refers to the contribution of GNDs to flow stress [160]. The long-range back stress generated by GNDs will also increase the resistance of dislocation slip and cause strengthening. More importantly, the high back stress can lead to the formation of high-density mobile dislocation inside the grains, improving the work-hardening capability and, thus, the plasticity of the GNG metals [120,136,144,169,170,171]. As one of the typical heterogeneous structure metals, the strain-strengthening of GNG metals is closely related to the evolution of GNDs density [139]. The research from Ashby et al. showed that density of GNDs was inversely proportional to the grain size, but proportional to the plastic strain [169]. Meanwhile, the contribution of GNDs to strength and plasticity varies with their density; the higher the density, the higher the back stress, and, thus, the higher the strengthening and work-hardening roles of the metals.

Although GNDs are proportional to the strain gradient, the quantitative relationship between strain gradient and GNDs is less studied. In particular, the quantitative relationships between the density of GNDs and mechanical properties all need further clarification. In addition, the higher the density of GNDs, the higher the back stress, the higher the density of mobile dislocations formed inside the grain, and thus the higher the plasticity of the metals. In other words, is there also a specific relationship between GNDs and mobile dislocations inside the grains? The relationship between these two and the effect on plasticity also needs further investigation. Finally, the pile-up of GNDs can prevent strain concentration and, thus, strain localization in nanostructured metals, but the effect of the degree of pile-up on the interaction between GNDs and other types of dislocations still needs further clarification.

#### 4.3.3. The Relationship between GNDs and the Work-Hardening Rate

Work hardening refers to the phenomenon where the strength of a metal increases continuously with the increase of strain during deformation [160]. The GNG metals have excellent work-hardening capability, which is one important property distinguishable from the traditional homogeneous metals [137]. The work-hardening behavior can be quantified by the work-hardening rate (Θ):

(3) $$\Theta \;=\frac{d\sigma }{d\varepsilon }$$

where σ is the true stress and ε is the true strain. The ductility of a metal is closely related to the work-hardening rate. According to Hart’s rule [172], the condition for metals to start plastic deformation is as follows:

(4) Θ+m·σ ≤ σ

where m is the strain rate sensitivity. Generally, the m value is less than 0.05. In order to obtain good ductility, the work-hardening rate needs to be high enough to withstand the increased stress, otherwise the metal will undergo necking and unstable fracture. Therefore, according to Hart’s rule, a higher work-hardening rate is the premise to ensure stable plastic deformation and good ductility.

The work-hardening rate depends on the net accumulation velocity of dislocations and is regulated by multiplication and annihilation of dislocations [137]. Increasing the multiplication ability can effectively improve the work-hardening ability, thereby enhancing the plasticity. There are two main types of dislocations in GNG metals, intrinsic dislocations generated by external tensile stress and GNDs induced by coordinated strain gradients. The strain gradient can promote the generation of GNDs, i.e., increase the total dislocation density, and thus enhance the work-hardening ability and achieve excellent plasticity [160].

The GNT structure often not only includes the GNG structure but also the GNT structure; its strengthening and toughening mechanism has contributions not only from the GNG structure, as described earlier, but also from the GNT structure. During the plastic deformation of GNT metals, in addition to the work hardening caused by the GNDs, the twins inside the grains strengthen the metal by impeding dislocation movement and enhance the plasticity by absorbing dislocations, and thus further enhance the mechanical properties of GNT metals [28,68].

In summary, the gradient-structured (GNG and GNT) metals generate strain gradient during deformation due to YS gradient, which is generated by the size gradient of microstructure constituents, and GNDs are distributed at interfaces in order to coordinate the strain gradients. The GNDs increase the dislocation resistance through short-range dislocation interactions and long-range back stresses, thus improving the strength and plasticity of the metals. For the GNT structure, in addition to the strengthening and toughening role played by the GNG structure mentioned above, the interactions between twins and dislocations, impeding dislocation motion, and absorbing dislocation further improve the strength and plasticity, and have superior mechanical properties compared to GNG metals.

However, there are still many problems that need to be further clarified regarding the deformation mechanisms, such as the quantitative relationship between strain gradient and GNDs, the relationship between GNDs and mobile dislocations inside the grains, and the interactions between GNDs and different types of dislocations during the deformation process. These problems should be the focus of the next research.

### 4.4. Summary and Prospect

As a new type of nanostructure, the gradient-nanostructured metals have been rapidly developed in terms of preparation technology, comprehensive performance improvement, and deformation mechanisms. However, there are still many important problems that need to be further clarified:

(1) Controllable preparation technologies. At present, the preparation of gradient-nanostructured metals is still dominated by gradient plastic deformation, which can only construct a gradient-nanostructured thin layer on a metal surface, and the volume fraction of gradient structure is very limited, generally 20–25% of the whole sample. More importantly, it is also difficult to accurately control the structure constituents, such as the size and distribution of structure constituents, to achieve the desired microstructure. Meanwhile, similar to the DPD method, the obtained structure is not “clean” and contains high-density dislocations. In contrast, the deposition method can achieve gradient structure distribution by varying the process parameters; similar to the PVD method, the obtained samples have a “clean” microstructure and can fabricate metals with gradient structure from the surface to the interior. However, it is also difficult to realize the engineering application. It can be seen that the development of preparation techniques that can produce gradient structure with higher volume fraction and can accurately control the structure constituents is the focus of the next step in the development of gradient nanostructures, and how to obtain samples with low initial dislocation density is also a problem that needs to be solved.
(2) The relationship between structure and mechanical properties. This relationship is mostly qualitative because the structural gradient from nanometer to macroscopic cannot be precisely controlled during the preparation process, and thus the optimization of the gradient nanostructure is also empirical. This relationship essentially includes the quantitative relationship between strain gradient and GNDs, the specific relationship between GNDs and mobile dislocations, the relationship between the density of GNDs and mechanical properties, etc. Therefore, to understand the relationship between structure and mechanical properties, these relationships need to be clarified. Moreover, coupling of different structures to further improve the mechanical properties of nanostructured metals is only achieved in the GNT structure currently. Hence, how to couple different strengthening and toughening structures to produce nanostructured metals with better mechanical properties is also a scientific issue worthy of further discussion. Due to the limitations of controllable preparation technology, the development of reliable and accurate theoretical models and calculation methods to describe and predict the mechanical properties of metals coupled with different gradient microstructures, and to effectively guide experimental investigation by optimizing the design of microstructures, will be another focus of theoretical research on nanostructured metals.

## 5. Supra-Nano-Dual-Phase Nanostructure

### 5.1. Design Concept

Amorphization is an effective way to increase the strength of metals greatly due to the unique short-range ordered and long-range disordered atomic arrangement structure of amorphous metals. However, the strength of amorphous metals still cannot reach the ideal value of about E/10 (where E is the elastic modulus of amorphous metals). Moreover, unlike the crystalline metals, in which there are dislocations, GBs, and other crystalline defects that can carry deformation, the deformation of amorphous metals is strongly localized into nanoscale shear bands; the subsequent expansion of the shear bands eventually leads to a catastrophic fracture, and the maximum strain is always only ~2% [29,173,174].

Based on the theoretical basis that the amorphous phases effectively suppress the softening of nanocrystals and the nanocrystals suppress the strain localization due to shear deformation of amorphous phases, Lu et al. [67,68] proposed an SNDP nanostructure consisting of two phases with characteristic size below ~10 nm and excellent mechanical properties. The two phases are mainly composed of one or both of the following structures, including supra-nanocrystal (SNC), supra-nano metallic glass (SMG), glass–glass interfaces (GGIs), and secondary element/structure-enriched GB. Thus far, four typical SNDP structures have been observed experimentally. Type 1 is composed of SNC and SMG, i.e., SNC + SMG; type 2 includes SMG and GGIs with different atomic packing structures and/or compositions (SMG + GGIs); type 3 is constituted by SNC and secondary element/structure-enriched GB (SNC + GB); type 4 is composed of SNC and SNC, with different crystalline structure and/or composition (SNC_1_ + SNC_2_).

### 5.2. Research Progress

At present, SNDP metals are mainly prepared by deposition methods, such as magnetron sputtering and inert gas condensation method. Magnetron sputtering is a kind of PVD. For the inert gas condensation method, nanoscale metal crystal particles are first synthesized in an inert gas atmosphere, and then consolidated for several times under ultrahigh vacuum and high pressure to obtain nanostructured metals. The advantages of the above deposition methods include a wide range of applicable metals, controllable thickness, and good microstructure homogeneity of the achieved metals [175]. Although the thickness of the sample can be controlled, the thickness of the sample is mostly only in the micron range and the preparation efficiency is low.

**Figure 12:**
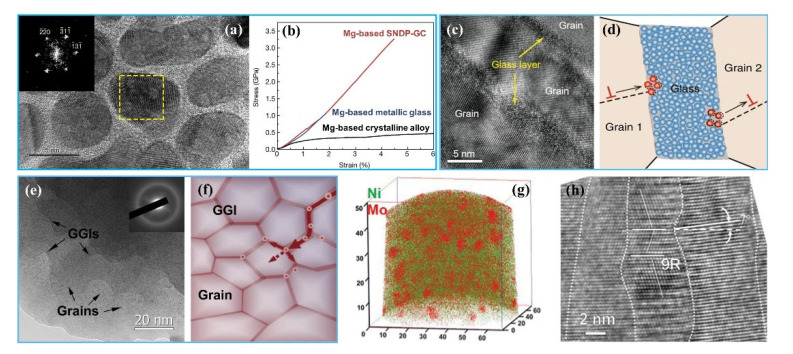
(**a**) HRTEM image of the Mg-based SNDP nanostructure, and the inset is the fast Fourier transform (FFT) image of the MgCu_2_ nanocrystal marked by the dashed line [29]; (**b**) engineering stress–strain curves for micropillar Mg-based SNDP glass crystal, a Mg-based metallic glass, and a Mg-based crystalline alloy [29]; (**c**) HRTEM image of nanostructured Al_95_Ni_2_Y_3_ alloy [30]; (**d**) schematic of dislocation activities [30]; (**e**) HRTEM image of the Sc–Fe supra-nano-glass, the inset is the SAED pattern recorded from the region shown in (**e**) [176]; (**f**) schematic of the GGIs in the supra-nano-glass (the arrows refer to the growth directions of embryonic shear band and the symbols represent the shear transformation zone) [176]; (**g**) APT image showing distributions of Ni and Mo in the Ni–Mo alloy sample [173]; (**h**) HRTEM image of the Al–Fe alloy showing high-density 9R phase, coherent TBs, and SFs [177].

Wu et al. [29] used magnetron sputtering to fabricate a Mg alloy thin film with an amorphous/nanocrystalline dual-phase structure (type 1, SNC + SMG), i.e., 56% volume fraction of MgCu_2_ nanograins with a diameter of ~6 nm, uniformly embedded in Mg-enriched amorphous Mg_69_Cu_11_Y_20_ matrix (Figure 12a). Its deformation ability was twice higher than that of Mg-based SMG, and the strength (~3.3 GPa) was ~9 times higher than that of the corresponding ultrastrong Mg-based crystalline alloy (Figure 12b), reaching an ideal strength E/20 of metallic glass and being very close to the theoretical strength (E/10). One strengthening and toughening mechanism was proposed based on constitutive modeling: a shear band was first generated and propagated in the amorphous shell during deformation, and its propagation was then blocked when the shear band encountered the MgCu_2_ nanocrystals, generating two sub-shear bands; then, numerous radiation-shaped embryonic shear bands were formed by the continuous increase of the stress and the impede effect of nanocrystals. Moreover, within any shear bands that do appear, embedded crystalline grains divide and rotate, contributing to hardening and countering the softening effect of the shear band. In this case, the conventional GB slip of nanograined Mg alloy and the resulting softening is replaced by the generation of multiple embryonic shear bands in the amorphous shell. In a word, the impeding of shear band propagation by nanocrystals greatly increases the strength compared to amorphous structures, while the division and rotation of nanocrystals induced by shear bands enhances plasticity.

Despite that the above study could provide ultrahigh strength yet loss of plasticity compared to the corresponding Mg-based crystalline alloy, this may be attributed to the brittle nature of the intermetallic MgCu_2_ nanocrystals. Therefore, Wu et al. [30] prepared a hierarchical nanostructured Al_95_Ni_2_Y_3_ alloy by substituting the brittle intermetallic phase with the ductile Al phase by magnetron sputtering. The structural unit of the sample was lamellar with ~40 nm width and ~100 nm length, the interfaces between the structural units were ~4 nm thick SMG phase (type 1, SNC + SMG) (Figure 12c), and the sample had a compression YS of ~1.7 GPa (tensile YS of ~1.2 GPa). During plastic deformation, the sample showed that the ultrahigh strength caused by the SMG phases preventing the slip of dislocations is initiated from the nanograin/SMG interfaces. Some dislocations pile up in the nanograins and provide strain hardening, while the majority of the dislocations move within the nanograins and are annihilated at another nanograin/SMG interface (Figure 12d). This continuous generation–movement–annihilation of dislocations in such a way led to a high plasticity (higher than ~70% when compressed, and up to ~15% under uniaxial tension).

The microstructure of supra-nano-glasses consists of nanoscale contiguous glassy regions (grains) and GGIs between these regions. A Sc_75_Fe_25_ supra-nano-glass sample (type 2, SMG + GGIs) was prepared by using the inert gas condensation method. Its structure was composed of glassy grains with a diameter of ~10 nm (hard zones) and GGIs with a thickness of ~1 nm (soft zones) (Figure 12e). The sample exhibited a YS of ~1.3 GPa and a plastic strain of ~18% under uniaxial tension, which was far greater than that of a metallic glass sample with similar size [176]. Later, a Cu_50_Zr_50_ supra-nano-glass (type 2, SMG + GGIs) was synthesized by using magnetron sputtering in an inert gas condensation system. In the structure, both Cu- and Zr-enriched glassy grain sizes were ~6 nm dispersed in Cu_50_Zr_50_ nano-glass matrix. The hardness and Young’s modulus of the sample were increased by ~11% and ~12%, respectively, compared to these of the melt-spun ribbon counterpart of the same composition [178]. More importantly, homogeneous deformation was observed in supra-nano-glass during indentation, whereas the melt-spun ribbons deformed by shear bands.

The atomic structure of amorphous alloy is loosely arranged and there are many excess pores, i.e., free volume, between atoms and atoms or atom clusters and atom clusters (Figure 12f) [176,179]. Supra-nano-glass (type 2) is a new type of amorphous alloy with heterogeneous microstructure; the heterogeneity is manifested in the difference in the densities of glassy grains and GGIs, which leads to a heterogeneous distribution of free volume. The density of GGIs is lower than that of glassy grains, i.e., higher free volume concentration, so they are more likely to become the shear band nucleation regions upon loading [180]. The embryonic shear band in the GGIs grows to a grain size close to that of the glassy grain and is then impeded by the harder grain with less free volume, and thus penetrates the grain or deflects the sliding to another GGIs [176]. This effectively imparts a strain-hardening mechanism and delays the catastrophic localization tendency seen in metallic glasses, and plastic flow will then occur in many soft zones, leading to a more uniform and enhanced plastic strain and, thus, superior mechanical properties.

Since softening behavior is usually attributed to GB sliding, can enhancing the stability of GBs avoid softening? Hu et al. [173] fabricated Ni–Mo alloy (type 3, SNC + GB) foil samples with Mo concentration in a range of 0.8 to 21.5 at.% using the direct current electrodeposition technique, in which Ni and Mo were uniformly distributed in the sample (Figure 12g). The grain size decreased with increasing Mo content, and the corresponding grain size changed from 16 to 2 nm when the Mo concentration increased from 0.8 to 21.5 at.%. Moreover, the sample exhibited significant annealing-induced hardening phenomena; the hardness increased from 5.02 GPa in the as-deposited state to 11.35 GPa in the as-annealed state, and the grain size was coarsened to ~8 nm. The microstructure of the annealed sample showed that the Mo was enriched at GBs and depleted inside the grains, and the GB excess energy was reduced by ~7 to 30% (similar to how the GB excess energy of Ni–12.7 alloy was reduced from 1.01 J/m^2^ to 0.70 J/m^2^ after annealing), which reduced the thermodynamic driving force for grain coarsening and improved the GB stability, making it difficult for the GB to slide under applied stress. Meanwhile, the dominant plastic deformation mechanism was changed from the GB-mediated process to generation of extended partial dislocations, while the massive nucleation of extended dislocations at the stabilized GBs required a very high applied stress, and thus obtained extremely high hardness. Such structure subverted the inverse Hall–Petch relationship and successfully eliminated the GB softening effect [68].

The 9R phase is an extended incoherent TB with periodically arranged SFs, and it has formation energy much higher than the nanotwin and is extremely unstable in Al [181]. However, Zhang et al. [177] found that the addition of Fe to Al improved the thermal and mechanical stability of 9R phase by MD simulations. Therefore, Al–Fe solid solution alloys with different Fe concentrations were prepared by direct current magnetron sputtering (type 4, SNC_1_ + SNC_2_), in which Fe and Al elements were uniformly distributed, and some of the columnar grains contained high-density nanotwins, and parallel-aligned 9R phase populated entire columns (Figure 12h). The grain sizes of the samples decreased with increasing Fe concentration, and the columnar grain diameter was 4 nm and 40 nm for Al–5.9 at.% Fe and Al–2.5 at.% Fe, respectively. The Al–5.9 at.% Fe had a hardness as high as 5.5 GPa and a flow stress exceeding ~1.5 GPa, and it tolerated more than 50% true strain without shear bands during compression. High density of SFs in 9R phase as pre-existing defects contributes to significant strengthening, and the Fe solute may retard incoherent TB migration and restrict dislocation transmission. MD simulations show that the 9R phase in Al–Fe impedes the glide of dislocations and thus strengthens the alloy, i.e., strain hardening, the hardening exponent of ~0.6, higher than that of ~0.3 for monolithic Al, subjected to compression, leading to a peak strength of nearly 6 GPa. Moreover, the interactions between 9R phases and dislocations lead to a significant distortion of the 9R phase, indicating the flexibility of the 9R phase, i.e., the 9R phase also makes significant contribution to plasticity while maintaining high strength. This research provides a new strategy for the design of ultrahigh-strength Al alloys.

In summary, the supra-nano metals are mainly prepared by magnetron sputtering and inert gas condensation methods. Although the above methods can prepare desirable structure, it is still difficult to prepare bulk samples and engineering applications. Comparing the above four types of SNDP structures, it is found that the enhancement of mechanical properties by SNC + SMG is much higher than the other three types, which may be attributed to the fact that the heterogeneity between SNC and SMG is much greater than that of other structures (such as glassy grains and GGIs). In addition, there are still some problems that remain unclear, such as the Mg [29] and Al [30] alloy in type 1; both are SNC + SMG structures, but their strengthening mechanism is nanocrystals hindering shear band propagation and SMG phase preventing dislocation slip, respectively. In addition, when the 9R phase appears in the Al–Fe alloy, the strengthening and toughening effect is much higher than that of nanotwins; is this related to the high SFE of Al? Are there other elements similar to Mo, that stabilize the GBs of Ni–Mo alloy, and Fe, that stabilize the 9R phase of Al–Fe alloy, and thus improve the mechanical properties? These problems need further investigation.

### 5.3. Summary and Prospect

The advantages of combining two heterogeneous phases in the supra-nano structure are that the supra-nano metals have good deformation ability while possessing high strength [49,182,183], and the supra-nano structure opens a new research idea for obtaining metals with excellent mechanical properties [68,178]. Thus far, magnetron sputtering and inert gas condensation method are the main preparation methods, but the preparation efficiency of these methods is also quite low and the thickness of the obtained samples is limited, which makes it difficult to apply them in engineering. Therefore, the development of efficient fabrication technology for the bulk SNDP metals will be the focus of the next research.

In addition, there are some problems that need to be solved. For example, investigating the following: why the deformation mechanisms in different metals with the same structure are not consistent; the relationship between the 9R phase and SFE; and the exploration of more elements that can stabilize interfaces or strengthening and toughening phases (such as GB or 9R phases), and thus improve mechanical properties. Moreover, studies on the effects of size and volume fraction of heterogeneous two-phase materials on mechanical properties are still in the exploratory stage.

## 6. Conclusions

In summary, this article reviews the research progresses of four typical kinds of new nanostructured metals with excellent synergy of strength and ductility that can effectively break through the limitation of strength–ductility trade-off of metals: bimodal nanostructure, NT/HNT structure, gradient nanostructure, and supra-nano structure. The preparation technologies, resulting microstructures and mechanical properties, and corresponding strengthening and toughening mechanisms of the four types of nanostructured metals are introduced. More importantly, it can be concluded that the preparation technologies, such as SPD, electrodeposition, DPD, SMAT, SMGT, and magnetron sputtering method, are relatively mature and can effectively prepare the new nanostructured metals. 

In terms of mechanical properties, the HNT structure has better performance than the NT structure due to its unique 3D nanotwin network that increases the interactions between dislocations and TBs (such as impeding and absorbing dislocations). Similarly, the GNT structure shows a greater improvement in mechanical properties than the GNG structure due to that it not only includes the contribution of the GNG structure, but also the GNT structure. The SNC + SMG structure has the greatest enhancement of mechanical properties among the SNDP structures, which may be due to its greater heterogeneity than other types of structures.

The proposed deformation mechanisms and related strengthening and toughening mechanisms can basically qualitatively interpret the excellent mechanical properties of these metals. This review provides a framework for the research on nanostructured metals with excellent combination of strength and ductility through the following three aspects: (1) the structure design of nanostructured metals and corresponding preparation technique; (2) the microstructures and mechanical properties of the resulting metals; and (3) the related strengthening and toughening mechanisms.

Based on the above discussion, it can be proposed that the following aspects should be further clarified:

(1) Structure design based on strengthening and toughening mechanisms

Among the four new nanostructured metals, strain gradient can be used to design nanostructured metals with heterogeneous microstructure constituents, such as bimodal nanostructure and gradient nanostructure. The strengthening and toughening mechanisms based on the interactions between nanotwins and dislocations can be used to design high-density nanotwins in metals, such as NT and HNT structure. Different phases with structural or compositional differences are used to design metals with near-ideal strengths, such as SNDP metals.

Therefore, it is essential to explore the microstructure that can provide excellent mechanical properties by deepening the understanding of the relationship between microstructures and strengthening and toughening mechanisms to provide guidance for the future structure design of high-strength and high-toughness nanostructured metals.

(2) Preparation techniques based on the desired structure

Currently, researchers are developing different technologies to prepare high-strength and high-toughness nanostructured metals, such as thermomechanical processing, DPD, SMRT, and shot peening based on plastic deformation, and physical/chemical deposition and inert gas condensation based on deposition processes. Plastic deformation has the advantages of simple equipment and large sample size, but the prepared samples often have high-density dislocations inside, and it is impossible to obtain a “clean” desired structure. The deposition processes allow the preparation of clean structures and can obtain microstructure constituents with different sizes through adjusting process parameters. However, most deposition methods are expensive and inefficient, they are limited to small-scale samples prepared in the laboratory and are more suitable for theoretical studies. Hence, from the perspective of the structure design, developing large-scale, effective, and low-cost preparation methods to prepare the desired structure is still an important frontier topic in academia and industry.

(3) Research methods for strengthening and toughening mechanisms

Due to the difficulty of preparing specific nanostructured metals, the difficulty of obtaining the clean intrinsic structure, and the scale limitation of the detection, accurate and clear mechanisms of strengthening and toughening cannot be obtained by experimental means alone and often need to be combined with computer simulations. First-principle and MD simulation are used to establish accurate theoretical models to describe and predict the mechanical properties of metals with different microstructures, and to obtain more excellent mechanical properties by optimizing the microstructure constituents and, thus, providing effective guidance for structure design. Therefore, combining experiments and simulations to investigate the strengthening and toughening mechanisms will be another focus of theoretical research on structural metals, and this provides opportunities and challenges for materials science researchers.

## Data Availability

Not applicable.

## Abbreviations

NT	Nanotwinned
TBs	Twin boundaries
SNDP	Supra-nano-dual-phase
GBs	Grain boundaries
SPD	Severe plastic deformation
YS	Yield strength
UTS	Ultimate tensile strength
SS	Stainless steel
TEM	Transmission electron microscopy
EBSD	Electron backscatter diffraction
IPF	Inverse pole figure
DBs	Deformation bands
FGs	Fine grains
GNDs	Geometrically necessary dislocations
HNT	Hierarchical nanotwinned
SFE	Stacking fault energy
DPD	Dynamic plastic deformation
PVD	Physical vapor deposition
SF	Stacking fault
λ	Twin thickness
SAED	Selected area electron diffraction
SEM	Scanning electron microscopy
SRX	Static recrystallization grains
STEM-HAADF	Scanning transmission electron microscope-high angle annular dark field
NG	Nanograins
DS	Dislocation structures
HRTEM	High-resolution TEM
MD	Molecular dynamics
TWIP	Twinning-induced plasticity
SMAT	Surface mechanical attrition treatment
λ1	Primary twin spacings
λ2	Secondary twin spacing
GNG	Gradient-nanograined
GNT	Gradient-nanotwinned
SMGT	Surface mechanical grinding treatment
SMRT	Surface mechanical rolling technique
IF	Interstitial free
SNC	Supra-nanocrystal
SMG	Supra-nano metallic glass
GGIs	Glass–glass interfaces
FFT	Fast Fourier transform
